# Flu Virus Attenuates Memory Clearance of *Pneumococcus* via IFN-γ-Dependent Th17 and Independent Antibody Mechanisms

**DOI:** 10.1016/j.isci.2020.101767

**Published:** 2020-11-04

**Authors:** Ning Li, Xin Fan, Meiyi Xu, Ya Zhou, Beinan Wang

**Affiliations:** 1 Key Laboratory of Pathogenic Microbiology and Immunology, Institute of Microbiology, Chinese Academy of Sciences, Beijing 100101, China; 2 University of Chinese Academy of Sciences, Beijing, 100101, China

**Keywords:** Immunology, Microbiology, Virology

## Abstract

Bacterial coinfection is a major cause of influenza-associated mortality. Most people have experienced infections with bacterial pathogens commonly associated with influenza A virus (IAV) coinfection before IAV exposure; however, bacterial clearance through the immunological memory response (IMR) in coinfected patients is inefficient, suggesting that the IMR to bacteria is impaired during IAV infection. Adoptive transfer of CD4^+^ T cells from mice that had experienced bacterial infection into IAV-infected mice revealed that memory protection against bacteria was weakened in the latter. Additionally, memory Th17 cell responses were impaired due to an IFN-γ-dependent reduction in Th17 cell proliferation and delayed migration of CD4^+^ T cells into the lungs. A bacterium-specific antibody-mediated memory response was also substantially reduced in coinfected mice, independently of IFN-γ. These findings provide additional perspectives on the pathogenesis of coinfection and suggest additional strategies for the treatment of defective antibacterial immunity and the design of bacterial vaccines against coinfection.

## Highlights

• Memory protection against bacteria was impaired in coinfection
• Memory Th17 response to bacteria was reduced by IAV-induced IFN-γ
• The Th17 reduction was caused by impeded Th17 proliferation and migration
• Bacteria-specific antibody was reduced in coinfection independent of IFN-γ

## Graphical Abstract

**Figure:**
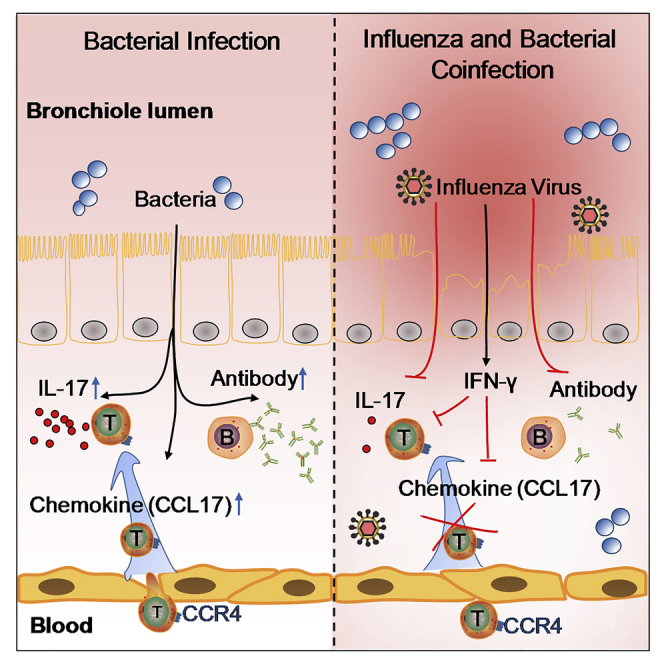

## Introduction

Influenza A virus (IAV) infection can render the host susceptible to bacterial coinfection, which is the leading cause of influenza-related death (McCullers, 2014; Morens et al., 2008). We have previously demonstrated that the IAV promotes the expression of host receptors, which facilitates bacterial adhesion to host cells and, consequently, efficient colonization (Li et al., 2015). Under normal conditions, bacterial colonization should be suppressed in immunocompetent hosts. Nevertheless, secondary bacterial infections can occur during viral clearance, suggesting that the immune response to IAV may lead to decreased immunity against bacterial infections (Brundage, 2006). Indeed, early innate responses against bacteria have been shown to be compromised as a result of a preceding viral infection (McCullers, 2014).

T-helper (Th)17 is an important T-cell subset induced by pathogenic bacteria at mucosal sites. Th17 cells are required for protective immunity against these pathogens (Rathore and Wang, 2016) and can be generated from effector memory CD4^+^ T cells to confer rapid and efficient antibacterial immunity (van Beelen et al., 2007). Attenuation of Th17 cell responses resulting from a preceding IAV infection is an important component of the increased susceptibility to secondary bacterial pneumonia in mice (Kudva et al., 2011; Lee et al., 2017; Robinson et al., 2014, 2015). Most human populations have experienced multiple episodes of infection by the bacterial pathogens commonly associated with IAV before virus exposure. Additionally, these colonizing bacteria should have been cleared by the immunological memory response (IMR), which confers efficient immune protection. The incomplete clearance of secondary bacterial infection suggests that the bacteria-specific IMR is impaired during IAV infection. This idea is supported by a recent study showing that vaccination against pneumococcal infection was highly efficacious in the absence of IAV exposure but only offered partial protection against secondary bacterial infections following IAV exposure (Metzger et al., 2015; Smith and Huber, 2018). Understanding the impact of IAV on the IMR to coinfecting bacteria could provide strategies to reduce disease severity and increase survival, as well as increase vaccine efficacy.

Interferon-gamma (IFN-γ) expression is induced in response to viral infection and is critical for immunity against viral and bacterial infections. Studies have indicated that IFN-γ is responsible for the impaired bacterial clearance during IAV infection (Duvigneau et al., 2016; Harada et al., 2016; Sun and Metzger, 2008); however, how IFN-γ affects the IMR to bacterial infection remains unknown. We hypothesized that memory Th17 cell responses to bacteria are impaired in the presence of high levels of IFN-γ induced by IAV, leading to inefficient bacterial clearance. T-cell migration is essential for T-cell responses (Groom, 2019; Krummel et al., 2016). Unlike naive T cells that predominantly traffic to secondary lymphoid organs, memory T cells exhibit higher expression levels of chemokine receptors, which enables them to infiltrate infected nonlymphoid tissues through interactions between the chemokine receptors and their chemokine ligands (Bromley et al., 2008; Fu et al., 2013). Chemokine receptor 4 (CCR4), a major trafficking receptor expressed on memory Th17 cells, is required for their migration into the lungs through chemoattraction to its ligand, CCL17, which is highly expressed on epithelial and endothelial cells of the lungs, as well as on dendritic cells (DCs) (Mikhak et al., 2013). Maintaining sufficient numbers of memory Th17 cells in the lungs is required for efficient clearance of reinfecting bacteria (Stolberg et al., 2011).

The aim of this study was to define the mechanisms underlying the reduced immune-related clearance of bacteria after IAV infection. We showed that bacterial clearance based on immunological memory was impaired in coinfected mice. The underlying mechanism was linked to the IFN-γ-mediated impairment of memory Th17 cell activation and migration to the lungs. In addition, the antibody-mediated memory response to *Streptococcus pneumoniae* (*Sp*) was inhibited in coinfected mice, independently of IFN-γ. These findings reveal new perspectives on the mechanisms of coinfection, and interventions targeting these mechanisms may help to lower the risk and severity of bacterial pneumonia after IAV infection.

## Results

### Memory-Mediated Bacterial Clearance Was Impaired in Coinfected Mice which Showed a Reduced Th17 Cell Response to Secondary *Sp* Infection

**Figure 1:**
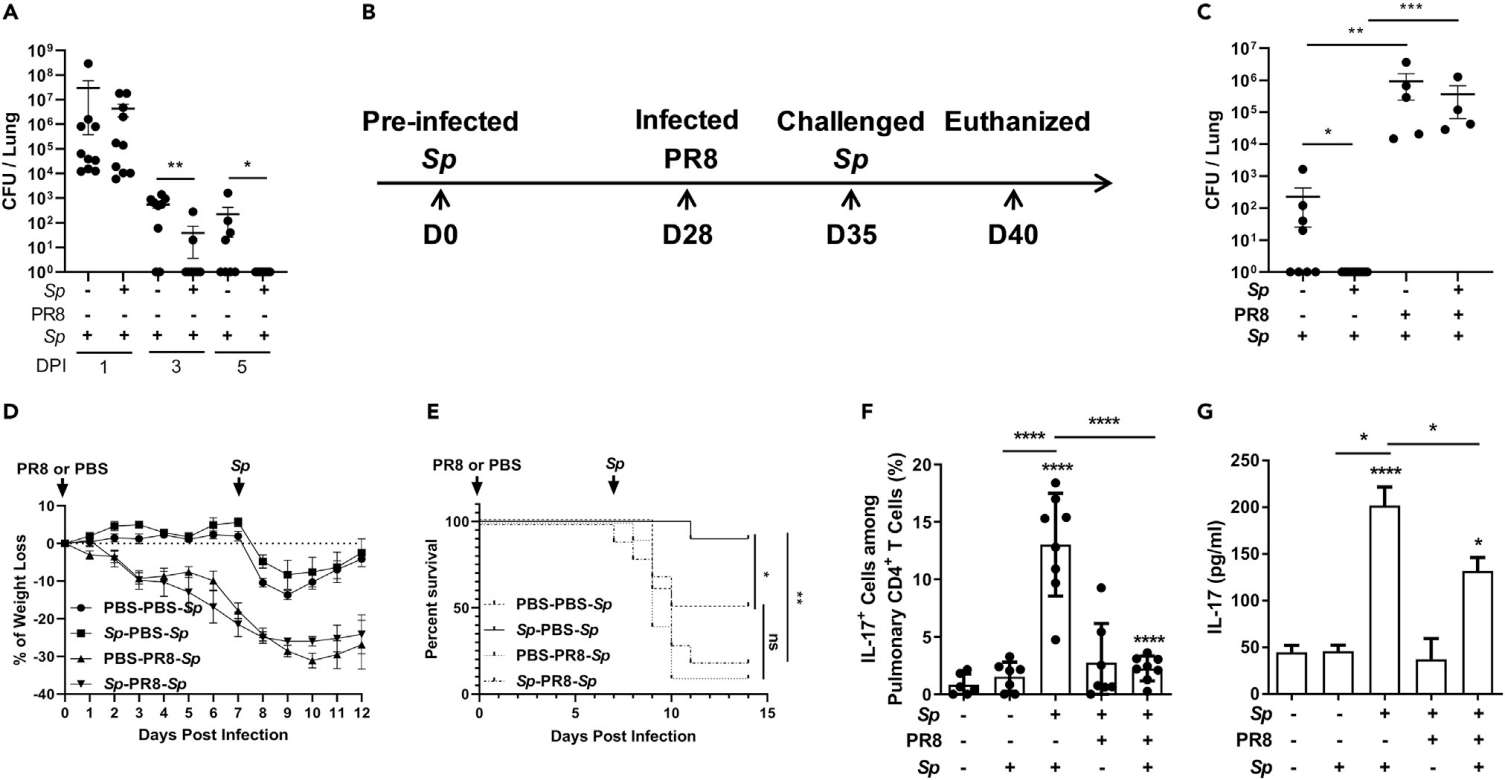
Memory-Mediated Bacterial Clearance Was Impaired in Coinfected Mice which Showed a Reduced Th17 Cell Response to Secondary *Sp* Infection (A) Mice were intratracheally (i.t.) inoculated with *Streptococcus pneumoniae* (*Sp*) and then challenged with *Sp* 5 weeks later. The mice were euthanized 1, 3, or 5 days after challenge for the determination of the numbers of colony-forming units (CFUs) in the lungs. (B) Schematic illustration of the experimental design. Mice were inoculated (i.t.) with *Sp.* Four weeks later, the mice were infected (i.t.) with the influenza A virus PR8 strain and challenged with *Sp* 7 days after infection. Five days after challenge, the mice were euthanized, and samples were taken for analyses. (C) The numbers of CFUs in the lungs were determined 5 days after challenge (*n* = 4–9). (D) Body weight was measured once daily (*n* = 4). (E) Mice were infected as in (B) but were challenged with a high dose of *Sp* following PR8 infection. The mortality rate was recorded daily (*n* = 10). Mice were infected, challenged, and euthanized as described in (B). (F) The proportion of IL-17^+^ cells among pulmonary CD4^+^ T cell population was determined by flow cytometry (*n* = 6–8). (G) IL-17 concentration in lung homogenates was determined by ELISA (*n* = 6–8). Data are represented as mean ± SEM of 2–3 independent experiments. ∗p < 0.05, ∗∗p < 0.01, ∗∗∗p < 0.001, ∗∗∗∗p < 0.0001. (A and C) Two-tailed unpaired Mann-Whitney *U* nonparametric *t* test; (E) log rank test; (F and G) one-way ANOVA, followed by Tukey's multiple comparisons test.

Mice were intratracheally (i.t.) inoculated with *Sp* or phosphate-buffered saline (PBS). After 5 weeks, the mice were reinfected with *Sp*, and bacterial colony-forming units (CFUs) in the lungs were determined 1, 3, and 5 days after reinfection. One day after *Sp* challenge, the number of CFUs was similar between PBS control and *Sp*-preinfected mice. However, substantially, fewer CFUs were recovered from *Sp*-preinfected mice compared with those treated with PBS at 3 days. Moreover, no CFUs could be recovered from *Sp*-preinfected mice after 5 days, whereas up to 1 × 10^3^ CFUs were detected in mice from the PBS group. This demonstrated that *Sp* preinfection established a memory response in the mice, which led to more efficient clearance of the infection (Figure 1A). To determine the impact of IAV infection on *Sp*-reactive memory T-cell responses, mice were first infected with *Sp*; 4 weeks later, the mice were either infected (i.t.) with a sublethal dose of the IAV PR8 strain or inoculated with PBS as a control and then challenged with the same dose of the *Sp* strain 7 days later (Figure 1B) (McNamee and Harmsen, 2006). Five days after *Sp* challenge, no CFUs were detected in the lungs of *Sp*-PBS-*Sp*-challenged mice. In contrast, ~1 × 10^5^ CFUs were detected in the PR8-preinfected mice (*Sp*-PR8-*Sp*) (Figure 1C); furthermore, mice infected with PR8 exhibited a ~20% loss of body weight 7 days after PR8 infection and another ~5% two days after the *Sp* challenge (Figure 1D). These results suggested that PR8 infection inhibited the IMR to *Sp*. To determine the mortality rate of coinfected animals, after PBS inoculation or *Sp* infection, the mice were challenged with a high dose of *Sp* following PR8 infection (PBS-PR8-*Sp* or *Sp*-PR8-*Sp*) or PBS inoculation (PBS-PBS-*Sp* or *Sp*-PBS-*Sp*). In total, 90% of the mice in the *Sp*-PBS-*Sp* group survived, whereas only 50% of the mice in the PBS-PBS-*Sp* group survived, demonstrating the protective effect of the IMR against *Sp*. Additionally, 90% of the mice in the *Sp*-PR8-*Sp* group and 80% of the mice in the PBS-PR8-*Sp* died (Figure 1E), suggesting that IMR to *Sp* was impaired following IAV infection, resulting in insufficient bacterial clearance and increased lethality.

The response of Th17 cells is critical for controlling *Sp* infection (Ramos-Sevillano et al., 2019). Flow cytometry analysis of lung cells revealed that, following *Sp* challenge, the number of CD4^+^ IL-17^+^ cells was substantially increased in the lungs of mice from the *Sp*-PBS-*Sp* group compared with that in mice from the *Sp*-PR8-*Sp* group; additionally, in the latter, CD4^+^ IL-17^+^ cell numbers were similar to those seen in nonpreinfected mice (Figures 1F, S1A, and S1B). Enzyme-linked immunosorbent assays (ELISAs) showed that the pulmonary level of IL-17 in the *Sp*-PR8-*Sp* group was substantially reduced compared with that in the *Sp*-PBS-*Sp* group (Figure 1G). Th1 cells can be activated in response to bacterial infection but are not a major Th subtype in the defense against mucosal bacterial infection (Wang et al., 2010). Compared with that observed in naive mice, Th1 cells did not respond to primary or secondary *Sp* infection, and the number of these cells was similar between the *Sp*-PR8-*Sp* and *Sp*-PBS-*Sp* groups (Figures S1C–S1E). These results suggested that the IAV reduced the response of memory Th17 cells to *Sp*.

### The Severity of Coinfection Was Associated with an Increased IFN-γ Response to IAV Infection

**Figure 2:**
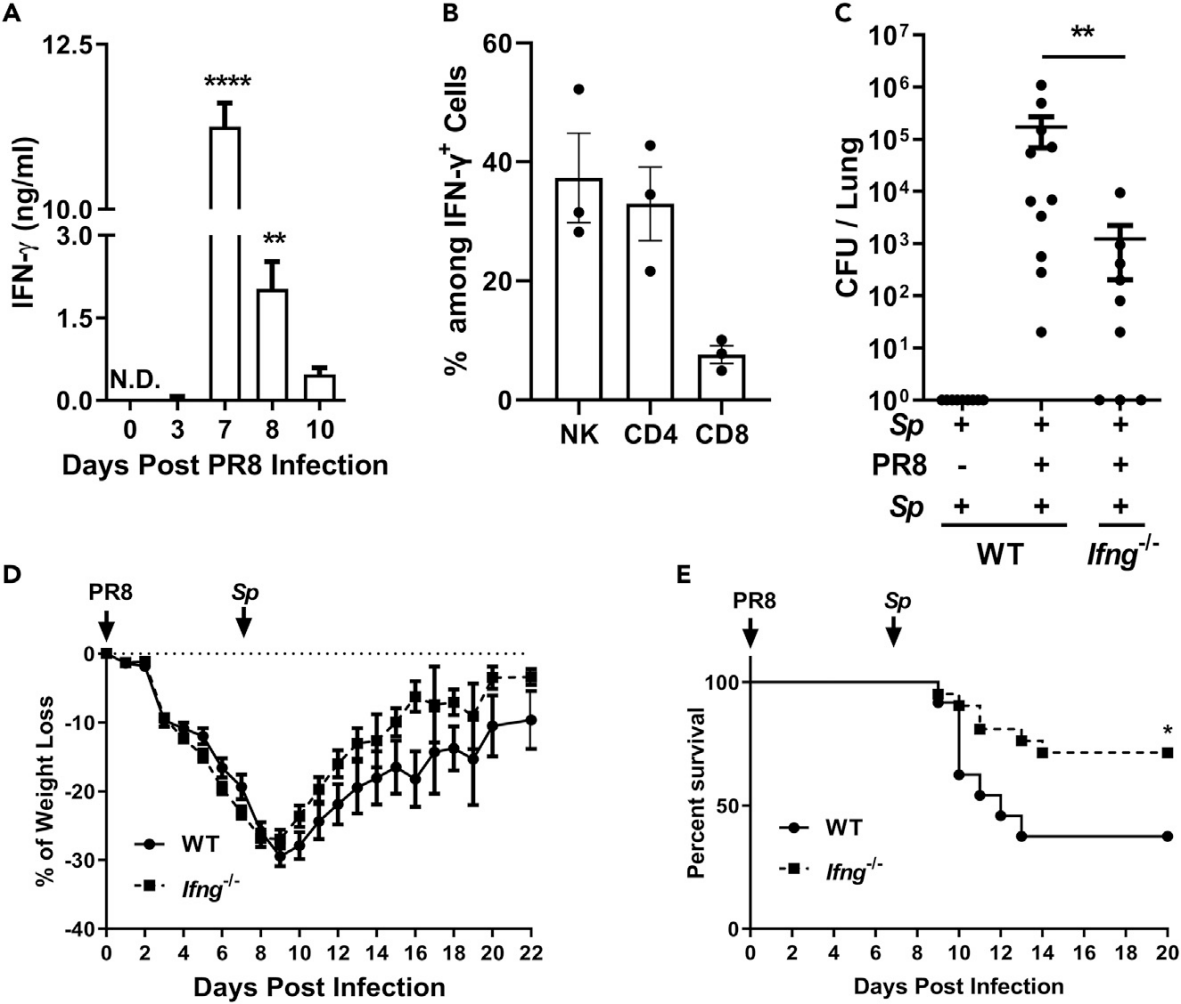
The Severity of Coinfection Was Associated with an Increased IFN-γ Response to IAV Infection (A) IFN-γ concentration in lung homogenates after infection with the influenza A virus PR8 strain was determined by ELISA (*n* = 4). (B) The proportion of CD4^−^ NK1.1^+^ cells, CD3^+^ CD4^+^ cells, or CD3^+^ CD8^+^ cells among IFN-γ^+^ cell population in the lungs was determined by flow cytometry 7 days after PR8 infection (*n* = 3). Mice were infected, challenged, and euthanized as described in Figure 1B. (C) The numbers of colony-forming units (CFUs) in the lungs were counted (*n* = 9–11). (D) Body weight was measured as described in Figure 1D (*n* = 6). (E) Mice were infected and challenged as described in Figure 1E. The mortality rate was recorded daily (*n* = 20). Data are represented as mean ± SEM of 2–3 independent experiments. ∗p < 0.05, ∗∗p < 0.01, ∗∗∗∗p < 0.0001. (A) One-way ANOVA, followed by Tukey's multiple comparisons test; (C) 2-tailed unpaired Mann-Whitney *U* nonparametric *t* test; (E) log rank test.

Mortality among coinfected mice has been reported to peak 6–7 days after IAV infection (McCullers and Rehg, 2002; McNamee and Harmsen, 2006). Here, we found that the pulmonary level of IFN-γ peaked 7 days after PR8 inoculation (Figure 2A) and that NK cells (37%) and CD4^+^ T cells (33%) were the main IFN-γ-secreting cells (Figure 2B), suggestive of a link between IFN-γ and disease severity. To verify this possibility, *Sp*-preinfected *Ifng*^*−*/−^ mice were inoculated with PR8 and challenged with *Sp* as described above. The number of CFUs recovered from the lungs 5 days after *Sp* challenge was ~100-fold lower in *Ifng*^*−*/−^ mice than in wild-type (WT) mice (Figure 2C), and *Ifng*^*−*/−^ mice displayed a faster recovery of body weight (Figure 2D). A lethality assay showed that >70% of the *Ifng*^*−*/−^ mice survived, compared with only 38% for the WT mice (Figure 2E). These results suggested that the increased IFN-γ level in response to IAV was linked to impaired memory-induced bacterial clearance.

### IFN-γ Deficiency Rescued the Response of Memory Th17 Cells to the Bacteria in Coinfected Mice

**Figure 3:**
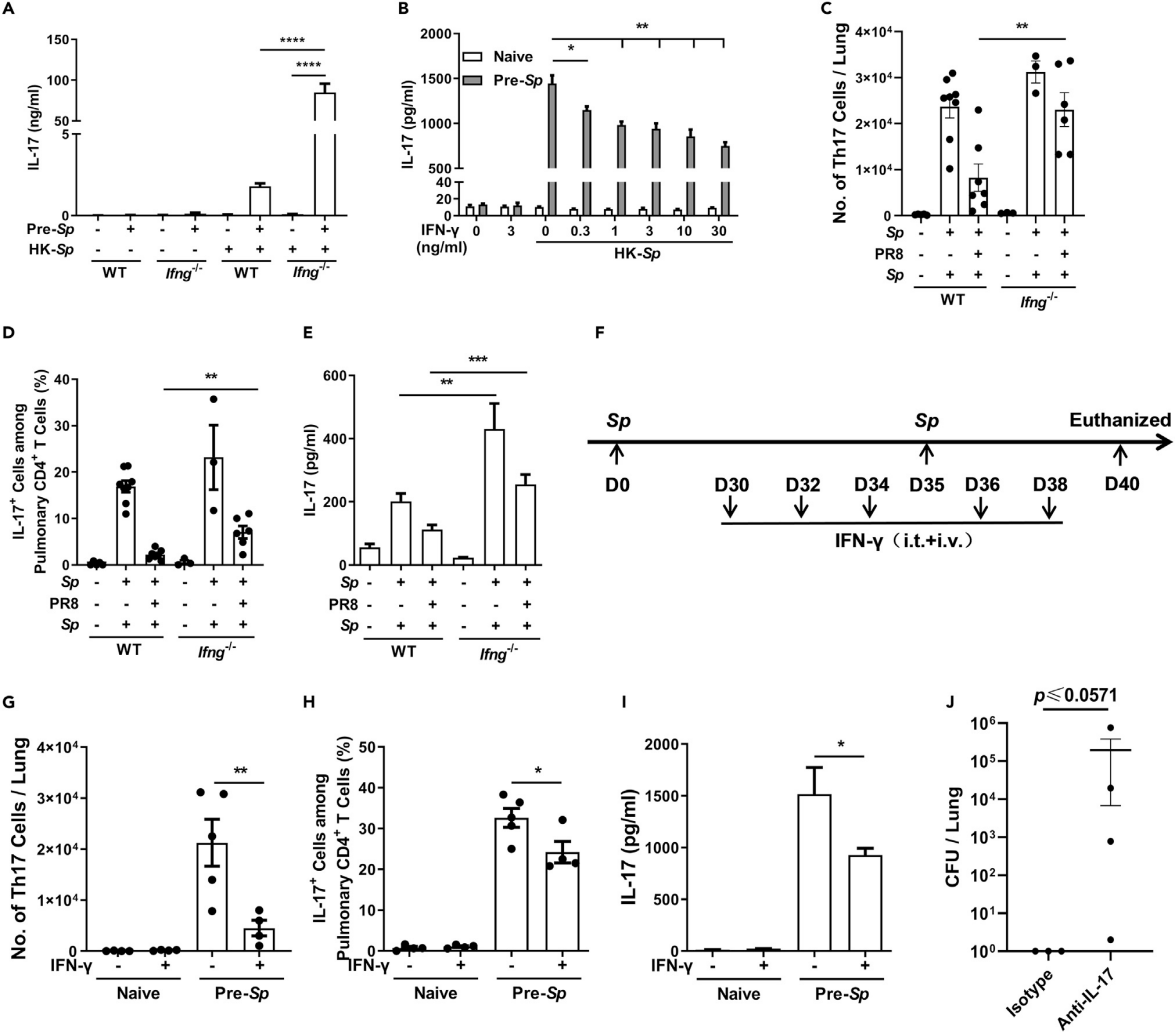
IFN-γ Deficiency Rescued the Response of Memory Th17 Cells to the Bacteria in Coinfected Mice (A and B) (A) Splenocytes from different groups of mice were cultured with or without heat-killed *Streptococcus pneumoniae* (HK-*Sp*) or (B) costimulated with the indicated concentration of recombinant mouse IFN-γ for 7 days (*n* = 6). IL-17 concentration in the culture supernatants was measured by ELISA. Mice were infected, challenged, and euthanized as shown in Figure 1B. (C and D) (C) The number of pulmonary IL-17^+^ CD4^+^ T cells and (D) the proportion of IL-17^+^ cells among CD4^+^ T cell population in the lungs were determined by flow cytometry (*n* = 3–8). (E) IL-17 concentration in lung homogenates was measured by ELISA (*n* = 6–8). (F) Schematic illustration of the experimental design for recombinant mouse IFN-γ treatment and infection in the mice. (G and H) (G) The number of pulmonary IL-17^+^ CD4^+^ T cells and (H) the proportion of IL-17^+^ cells among CD4^+^ T cell population in the lungs were detected by flow cytometry. (I) IL-17 concentration in lung homogenates was measured by ELISA. (J) *Ifng*^*−*/−^ mice were infected and challenged as described in Figure 1B and were intraperitoneally injected with neutralizing antibody directed against IL-17 (isotype antibody was used as a control) every other day after infection with the influenza A virus PR8 strain. The numbers of colony-forming units (CFUs) in the lungs were determined 5 days after challenge (*n* = 3–4). Data are represented as mean ± SEM of 2–3 independent experiments. ∗p < 0.05, ∗∗p < 0.01, ∗∗∗p < 0.001, ∗∗∗∗p < 0.0001. (A-E and G-I) One-way analysis of variance (ANOVA) A, followed by Tukey's multiple comparisons test; (J) 2-tailed unpaired Mann-Whitney *U* nonparametric *t* test.

Based on the correlation between IFN-γ induction and the low efficiency of bacterial clearance, we speculated that IFN-γ inhibits the response of memory Th17 cells to *Sp*. To test this, *Ifng*^*−*/−^ mice were preinfected with *Sp*, and the levels of IL-17 produced by the splenocytes were measured as an indicator of Th17 cell activation. ELISAs revealed that both WT and *Ifng*^*−*/−^ mice produced IL-17 in response to heat-killed *Sp* (HK-*Sp*) treatment; however, the levels of IL-17 were ~50-fold higher in *Ifng*^*−*/−^ mice than in WT mice (Figure 3A). To further determine the role of IFN-γ in the response of memory Th17 cells, splenocytes were isolated from *Sp*-preinfected WT mice and coincubated with IFN-γ in the presence of HK-*Sp*. ELISAs performed on the culture supernatant showed that the IL-17 recall response to HK-*Sp* was reduced in an IFN-γ dose-dependent manner (Figure 3B). Compared with WT mice, IL-17 production was much higher in splenocytes derived from *Sp*-preinfected *Ifng*^*−*/−^ mice and was inhibited more markedly by exogenous IFN-γ (Figure S2A). To confirm this observation, *in vivo* experiments were carried out. *Sp*-preinfected *Ifng*^*−*/−^ mice and WT mice were coinfected as described in Figure 1B, and the response of pulmonary Th17 cells was analyzed by flow cytometry*.* Higher numbers of CD4^+^ IL-17^+^ cells were detected in *Ifng*^*−*/−^ mice than in WT mice (Figures 3C, 3D, and S2B), and a similar propensity was observed for IL-17 production in lung homogenates obtained from *Ifng*^*−*/−^ mice (Figure 3E). To confirm these *in vivo* findings, *Sp*-preinfected *Ifng*^*−*/−^ mice were administered IFN-γ i.t. and intravenously to simulate IFN-γ induction by PR8 (Figure 3F). As expected, IFN-γ-treated mice displayed fewer pulmonary CD4^+^ IL-17^+^ cells and lower expression of IL-17 in response to *Sp* challenge (Figures 3G–3I and S2C). These data suggested that IFN-γ restricted the activation of memory Th17 cell responses. We noticed that the response of memory Th17 cells/IL-17 in *Ifng*^*−*/−^ mice of the *Sp*-PR8-*Sp* group was not completely reversed to the levels observed in WT mice of the *Sp*-PBS-*Sp* group, indicating that other mechanisms may be involved in the impaired memory Th17 cell response. To verify that the rescued memory Th17 cell response was responsible for the improved bacterial clearance under conditions of IFN-γ deficiency, *Ifng*^*−*/−^ mice were infected as described in Figure 1B and intraperitoneally injected with IL-17 neutralizing antibody following PR8 infection, following which the numbers of CFUs in the lungs of the mice were determined. The results revealed that, although statistical significance was not reached, mice that received IL-17 neutralizing antibody exhibited a greater bacterial load relative to those receiving the isotype control antibody (Figure 3J). These data demonstrated that PR8-induced IFN-γ inhibits the protection against *Sp* by suppressing memory Th17 cell responses.

### The Activation of DCs and IL-23 Produced by DCs Were Both Inhibited in IAV-Infected Mice Independently of IFN-γ

**Figure 4:**
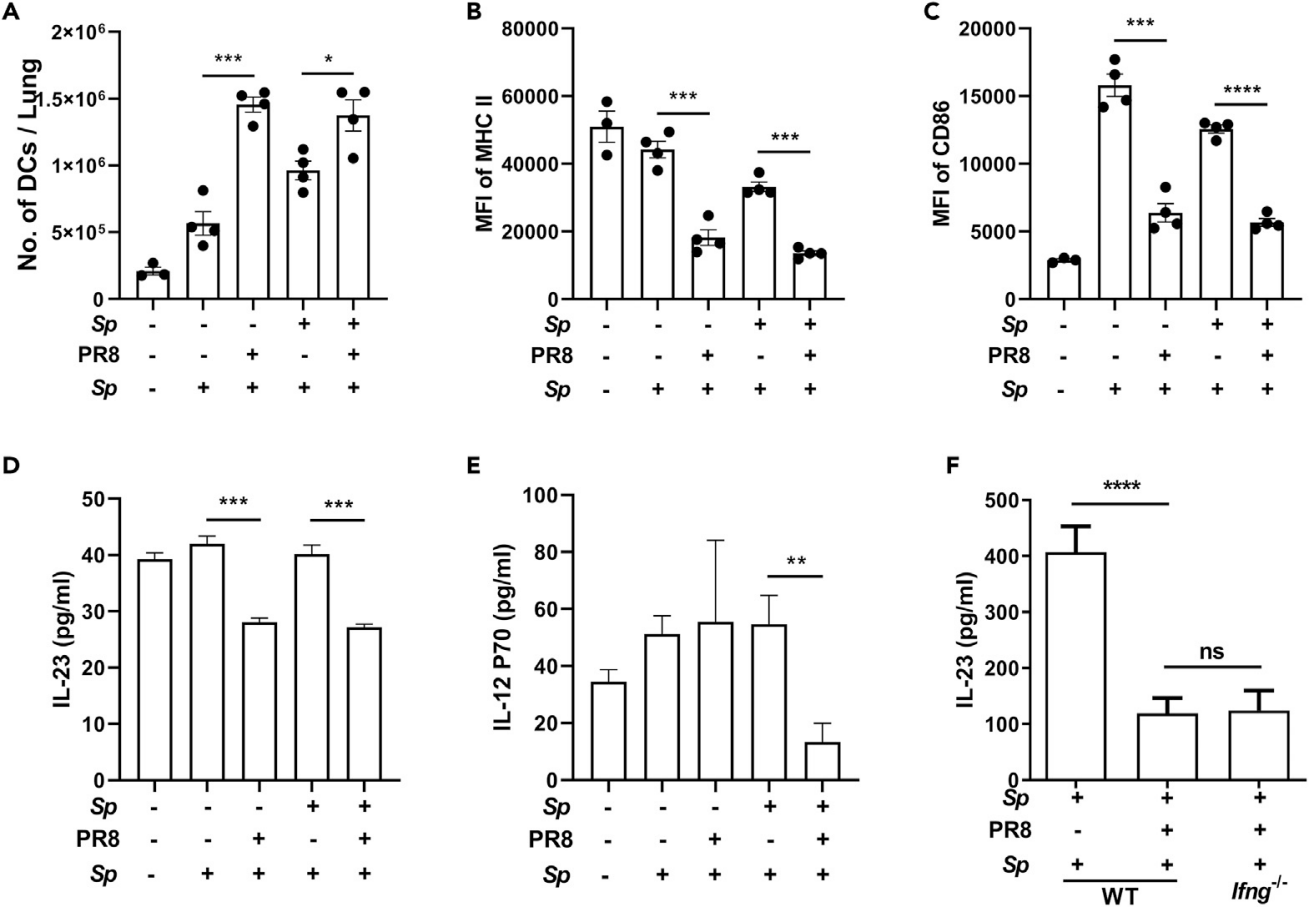
The Activation of DCs and IL-23 Produced by DCs Were Both Inhibited in IAV-Infected Mice Independently of IFN-γ Mice were infected and challenged as described in Figure 1B. Two days after *Streptococcus pneumoniae* (*Sp*) challenge, the mice were euthanized, and lung samples were taken for dendritic cell (DC) analyses (*n* = 4). (A–C) (A) The number of CD11c^+^ cells in the lungs and the mean fluorescence intensity (MFI) for (B) MHC class Ⅱ and (C) CD86 on the surface of DCs were determined by flow cytometry. (D and E) (D) IL-23 and (E) IL-12p70 concentration in lung homogenates was determined by ELISA. (F) Mice were infected, challenged, and euthanized as described in Figure 1B. IL-23 concentration in lung homogenates was determined by ELISA (*n* = 9–11). Data are represented as mean ± SEM of 2–3 independent experiments. ns, not significant, ∗p < 0.05, ∗∗p < 0.01, ∗∗∗p < 0.001, ∗∗∗∗p < 0.0001. (A-F) One-way ANOVA, followed by Tukey's multiple comparisons test.

DCs play a critical role in T-cell activation. To determine the mechanisms underlying the reduction in Th17 cell responses, we examined the effect of PR8 infection on DCs. Mice were infected as described in Figure 1B and euthanized 2 days after *Sp* challenge, the time when the activation of DCs reached the peak point (data not shown). We found that the levels of pulmonary DCs were substantially higher in PBS-PR8-*Sp*- and *Sp*-PR8-*Sp*-treated mice than in matching, PR8-uninfected controls (Figure 4A). Flow cytometry analysis revealed that the expression of MHC class Ⅱ and CD86 was lower in PR8-infected mice (Figures 4B, 4C and, S3A). DCs are a primary source of IL-23 secretion, and IL-23 is important for memory T-cell proliferation and IL-17 secretion (Li et al., 2019). ELISAs conducted on lung tissue supernatants showed that the levels of IL-23 were substantially reduced in PR8-infected mice irrespective of whether the mice had been preinfected or not (Figure 4D). The level of IL-12, a cytokine predominantly produced by activated DCs (Kaka et al., 2008), was also lower in the *Sp-*PR8-*Sp* treatment group than in the other groups (Figure 4E). Similar DC-related changes were also observed in the hilar lymph nodes (HLNs) (Figures S3B–S3F). These results suggested that DC activation was inhibited following PR8 infection. To determine the effects of IFN-γ on the DC-related changes, IL-23 production was examined 5 days after challenge. As shown in Figure 4F, much higher levels of IL-23 were found at this time (400 pg/mL) than those detected at 2 days (40 pg/mL) in *Sp*-PBS-*Sp* groups. However, the levels were equal between WT- and *Ifng*^*−*/−^-coinfected mice and much lower than those in *Sp*-PBS-*Sp* mice. These results indicated that the reduction in IL-23 levels observed in WT mice was not rescued in *Ifng*^*−*/−^ mice and supported that DC inactivation was IFN-γ independent.

It has been reported that IFN-γ inhibits memory Th17 cells through indoleamine 2,3-dioxygenase (IDO) produced by antigen presenting cells (APCs) in a mouse model of collagen-induced arthritis (Lee et al., 2013). To test whether the same mechanism was involved in Th17 cell inhibition, coinfected mice were administered the IDO inhibitor 1-methyl-D-tryptophan in drinking water before, during, and after PR8 infection (Figure S4A). We found that the percentages and numbers of pulmonary Th17 cells were similar between IDO inhibitor- and vehicle control-treated mice (Figures S4B and S4C). Moreover, no differences in the numbers of lung-derived CFUs or body weight loss were found between the two groups of mice (Figures S4D and S4E). These data indicated that the IFN-γ-dependent IDO pathway found in the non-infection model was not involved in the impairment of Th17 cell responses observed during coinfection.

### The Proliferation of Th17 Cells in Response to Secondary *Sp* Infection Was Suppressed in IAV-Infected Mice

**Figure 5:**
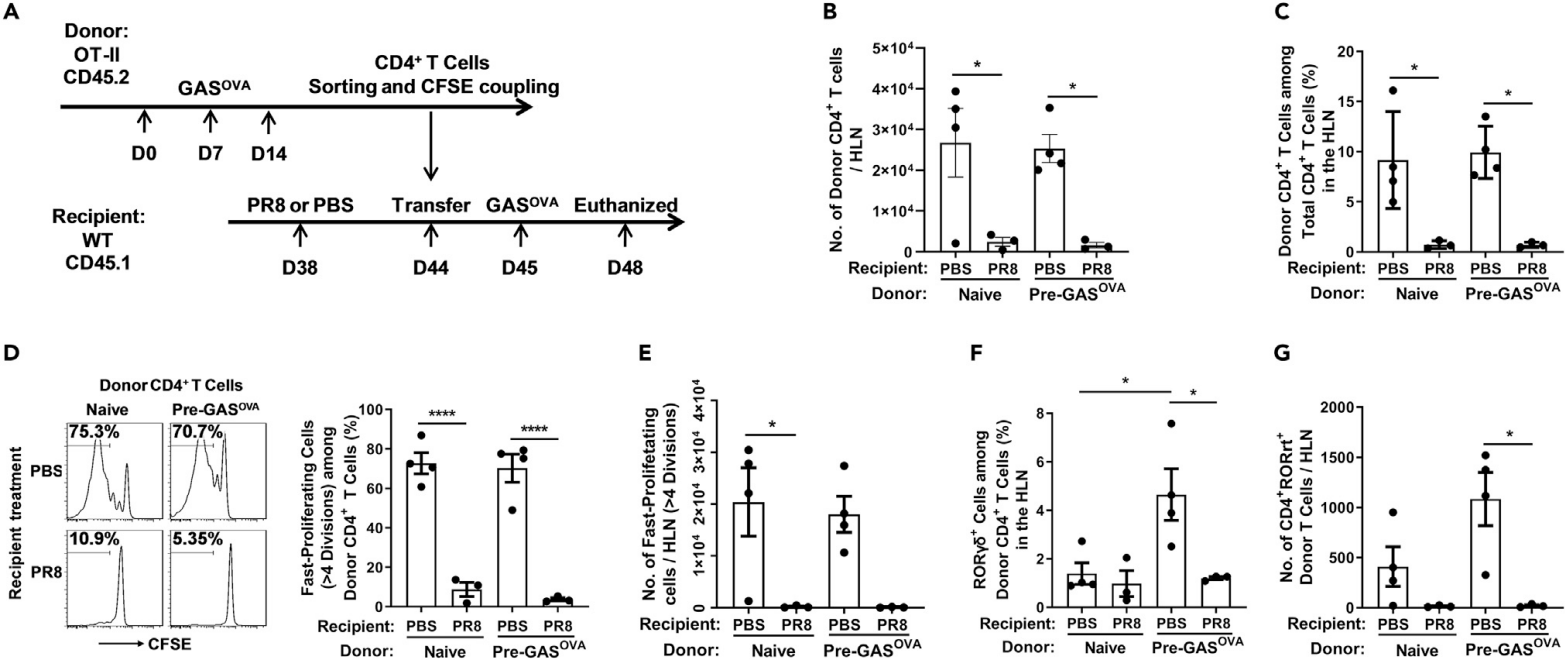
The Proliferation of Th17 Cells in Response to Secondary *Sp* Infection Was Suppressed in IAV-Infected Mice (A) Schematic illustration of adoptive transfer and mouse infection. The hilar lymph nodes (HLNs) of the recipients were removed for flow cytometric analyses. (B–G) (B) The number of donor CD4^+^ T cells and (C) the proportion of donor CD4^+^ T cells among the total CD4^+^ T cell population; (D) the proportion of fast-proliferating cells among the donor CD4^+^ T cell population and (E) the number of fast-proliferating cells; and (F) the proportion of RORγt^+^ cells among the donor CD4^+^ T cell population and (G) the number of RORγt^+^ CD4^+^ donor T cells were determined by flow cytometry. Data are represented as mean ± SEM of 2–3 independent experiments. ∗p < 0.05, ∗∗∗∗p < 0.0001. (B–G) One-way ANOVA, followed by Tukey's multiple comparisons test.

To analyze the mechanisms underlying the reduction in the number of Th17 cells, OT-Ⅱtransgenic mice (CD45.2) were preinfected with a previously generated *Streptococcus pyogenes* strain that expresses the ovalbumin 323–339 peptide (GAS^OVA^) (Park et al., 2004). *Streptococcus pyogenes* is a bacterium that is also frequently associated with coinfection. Four weeks after infection, CD4^+^ T cells were isolated from infected mice or naive OT-Ⅱtransgenic mice, stained with carboxyfluorescein succinimidyl ester, and transferred to recipient mice (CD45.1) that had been infected with PR8 6 days previously. Twenty-four hours after transfer, the recipient mice were infected (i.t.) with GAS^OVA^ (Caucheteux et al., 2017) (Figure 5A). Cells in the HLNs of the recipient mice were analyzed 3 days after GAS^OVA^ infection. Flow cytometry analysis revealed that the number of CD4^+^ CD45.2^+^ donor cells derived from GAS^OVA^-preinfected mice was lower in PR8-infected recipients than in those treated with PBS (Figures 5B, 5C, and S5A). A similar reduction in the number of donor cells was observed in PR8-infected recipients when naive donor cells were used, suggesting that PR8 infection inhibited the proliferation of both primary and memory T cells. Flow cytometry analysis of HLN cells revealed that the numbers of fast-dividing (>4 times) donor cells (Figures 5D and 5E) and CD4^+^ CD45.2^+^ T cells expressing RORγt^+^ (a lineage-defining transcription factor) in PR8-infected recipients were also substantially lower than those in PBS-treated recipients (Figures 5F, 5G, and S5B). These data suggested that PR8 infection inhibited the proliferation of CD4^+^ T cells, especially Th17 cells, in response to secondary bacterial infection.

### The Trafficking of Th17 Cells to the Lungs Was Delayed in Coinfected Mice in Response to Secondary *Sp* Infection

**Figure 6:**
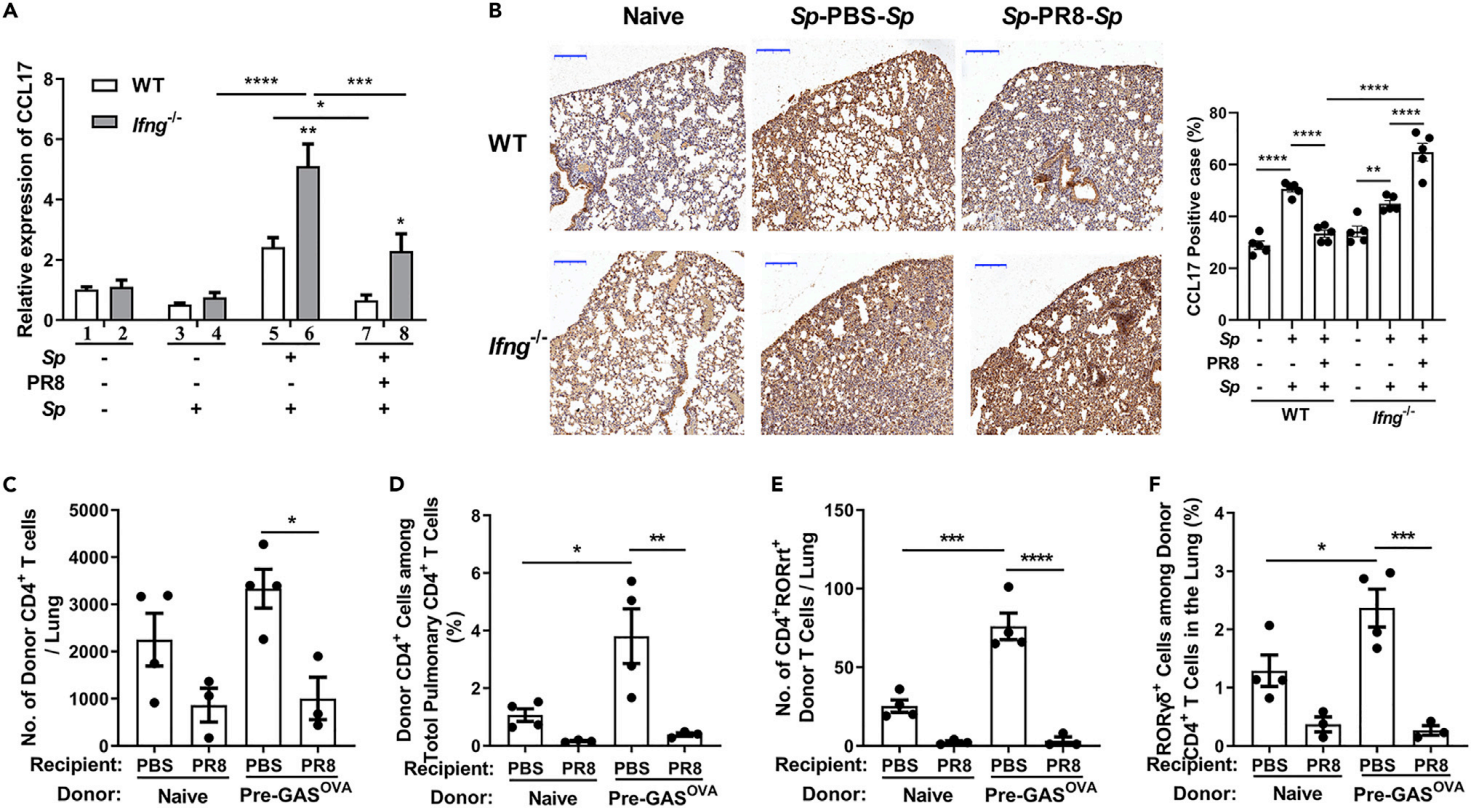
The Trafficking of Th17 Cells to the Lungs Was Delayed in Coinfected Mice in Response to Secondary *Sp* Infection Mice were infected, challenged, and euthanized as described in Figure 1B. (A) The relative expression of CCL17 in lung cells was determined by qPCR (*n* = 6–8). (B) Immunohistochemical staining of lung sections using an anti-CCL17 antibody. Scale bar represents 200 μm (left). The positive rate of CCL17 expression cells was assessed (right). The adoptive transfer was performed as described in Figure 5A. The lungs of recipients were removed for flow cytometric analyses (*n* = 3–4). (C–F) (C) The number of donor CD4^+^ T cells and (D) the proportion of donor CD4^+^ T cells among the total CD4^+^ T cell population and (E) the number of RORγt^+^ CD4^+^ donor T cells and (F) the proportion of RORγt^+^ cells among the donor CD4^+^ T cell population were determined by flow cytometry. Data are represented as mean ± SEM of 2–3 independent experiments. ∗p < 0.05, ∗∗p < 0.01, ∗∗∗p < 0.001, ∗∗∗∗p < 0.0001. (A–F) One-way ANOVA, followed by Tukey's multiple comparisons test.

The homing of T cells to specific tissues is crucial for evoking a robust immune response in infected sites (Tufail et al., 2013). CCR4 is a major trafficking molecule expressed on Th17 cells that is required to guide their recruitment to the lungs *via* CCL17 (Matsuo et al., 2016; Mikhak et al., 2013). To ascertain whether the reduction in the number of Th17 cells in the lungs was due to reduced T-cell migration, quantitative real-time PCR (qPCR) was carried out. The pulmonary expression of CCL17 was similar between *Sp*-preinfected mice and naive mice (Figure 6A, columns 1 and 3) and was substantially increased in mice from the *Sp*-PBS-*Sp* group (Figure 6A, column 5). In contrast, CCL17 expression remained at a basal level in *Sp*-PR8-*Sp*-coinfected mice (Figure 6A, column 7). Meanwhile, the CCL17 expression pattern was similar in *Ifng*^*−*/*−*^ mice but was much higher in response to *Sp* challenge (Figure 6A, gray columns). Further analyses of CCL17 expression in lung tissue by immunohistochemistry (Figure 6B, left) and the subsequent quantification of staining intensity (Figure 6B, right) revealed that the lower CCL17-positive rate in the *Sp*-PR8-*Sp* group was relative to that in the *Sp*-PBS-*Sp* group. The positive rate was markedly higher in *Ifng*^*−*/*−*^ mice than in WT mice following *Sp*-PR8-*Sp* treatment. These results suggested that IFN-γ contributed to the reduction in CCL17 expression in the lungs of coinfected mice. We also examined the levels of CCR4 on CD4^+^ T cells. Flow cytometric analysis revealed that the percentile of pulmonary CD4^+^ CCR4^+^ cells in *Sp*-PR8-*Sp*-treated *Ifng*^*−*/*−*^ mice was as high as that in *Sp*-PBS-*Sp*-treated WT mice (Figure S6A, columns 5 and 8) and that in *Sp*-PR8-*Sp*-coinfected WT mice was lower (Figure S6A, column 7). However, the numbers of CD4^+^ CCR4^+^ cells were similar between WT and *Ifng*^*−*/*−*^ mice (Figure S6B). These data suggested that IFN-γ restricts the migration of Th17 cells to the lungs mainly through the downregulation of CCL17 expression in coinfected lung tissue. To further verify this, CD4^+^ T cells from GAS^OVA^-infected OT-Ⅱtransgenic mice were transferred to PR8-infected recipient mice. Flow cytometry revealed that, after challenge with GAS^OVA^, the number and percentile of CD4^+^ CD45.2^+^ donor cells in the lungs of PR8-infected GAS^OVA^ recipients were significantly lower than those in the lungs of PBS-treated GAS^OVA^ recipients (Figures 6C, 6D, and S6C). Similar results were found when RORγt^+^ donor cells in the lungs were examined (Figures 6E, 6F, and S6D). These results suggested that IAV infection impeded the trafficking of Th17 cells to the lungs in response to secondary bacterial infection.

### Memory Antibody Responses to *Sp* Were Reduced in Coinfected Mice Independently of IFN-γ

**Figure 7:**
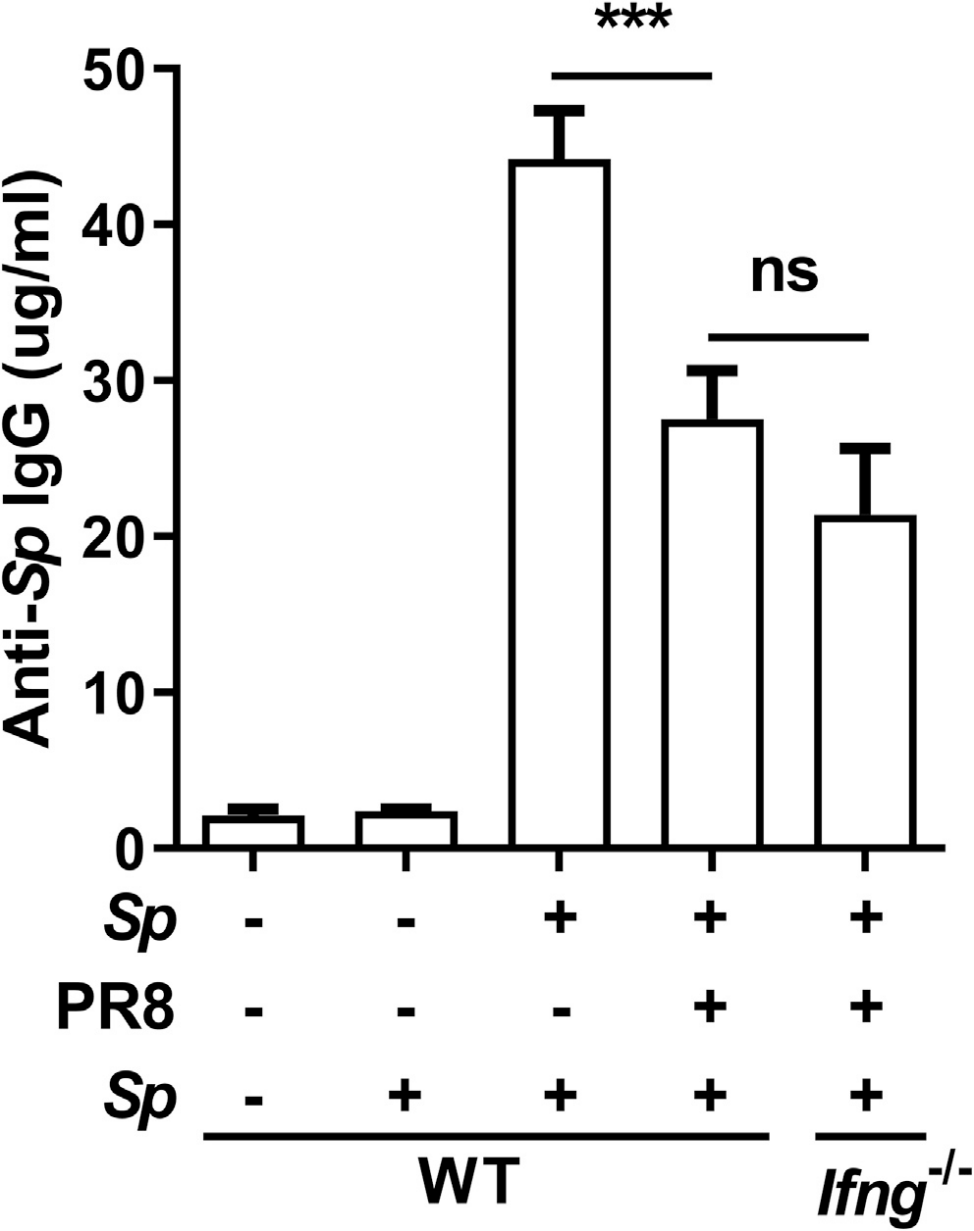
Memory Antibody Responses to *Sp* Were Reduced in Coinfected Mice Independently of IFN-γ Mice were infected, challenged, and euthanized as described in Figure 1B. *Streptococcus pneumoniae* (*Sp*)-specific IgG concentrations in serum were determined by ELISA (*n* = 6–12). Data are represented as mean ± SEM of 3 independent experiments. ns, not significant, ∗∗∗p < 0.001. One-way ANOVA, followed by Tukey's multiple comparisons test.

The *Sp* recall clearance was partially rescued in coinfected *Ifng*^*−*/*−*^ mice, suggesting that other, IFN-γ-independent mechanisms were involved. The antibody-mediated memory response is important for the elimination of re-entry pathogens through opsonophagocytosis and neutralization. Therefore, we measured the serum levels of *Sp*-specific IgG using ELISAs. IgG production was strongly induced in response to *Sp* challenge in the *Sp*-PBS-*Sp* group but markedly inhibited in the *Sp*-PR8-*Sp* group (Figure 7). However, different from the response of Th17 cells, IgG production was not rescued in coinfected *Ifng*^*−*/−^ mice (Figure 7), suggesting that IAV infection inhibited the memory antibody response to *Sp* through IFN-γ-independent mechanisms.

## Discussion

Immunological memory is long lived and responds rapidly and effectively to previously encountered pathogens (Janeway, 2001). Given that most human populations have experienced respiratory infections with the bacterial pathogens commonly found in IAV coinfection, the inefficient bacterial clearance during coinfection is likely attributable to an impaired IMR to the invading bacteria.

In the present study, we found that the responses by memory Th17 cells and antibodies to pneumococcal infection were attenuated during IAV infection, leading to impaired bacterial clearance and increased lethality. We demonstrated that the impaired memory Th17 response resulted from an IFN-γ-dependent reduction of Th17 proliferation and lung trafficking of Th17 cells and that the attenuated antibody recall response to bacterial infection was IFN-γ independent.

IFN-γ is a multipotent cytokine responsible for the modulation of many facets of the immune response (Zha et al., 2017). Although we demonstrated that the restriction of memory Th17 cells played an important part in bacterial clearance, other IFN-γ-dependent mechanisms may also have contributed, such as the inhibition of alveolar macrophages during bacterial clearance (Sun and Metzger, 2008). In addition, the partially reversed memory-mediated bacterial clearance and CCL17 expression in the lungs of *Ifng*^*−*/*−*^ mice also suggest that other IFN-γ-independent mechanisms are involved. Studies have shown that IAV infection can induce IFN-γ-independent DC differentiation defects in the bone marrow (Beshara et al., 2018), while type I IFNs can attenuate Th17 response *via* the suppression of IL-23 production by DCs (Kudva et al., 2011). Similarly, we found that IAV infection inhibits the activation of lung DCs and decreases IL-23 production by DCs independently of IFN-γ. Given the critical role of IL-23 in the regulation of memory Th17 cell function, the impaired activation of DCs during IAV infection might be responsible for the residual IFN-γ-independent impairment of *Sp* clearance, along with other mechanisms such as the type I IFN-mediated inhibition of neutrophil and macrophage chemotaxis (Nakamura et al., 2011; Shahangian et al., 2009) and restriction of IL-17-producing γδ T cells (Li et al., 2012).

Although IFN-γ has been shown to downregulate Th17 cell differentiation *in vitro* (Harrington et al., 2005; Nakae et al., 2007), similar results have not been evidenced *in vivo*. Mouse experiments have shown that a reduced Th17 response to bacterial infection in IAV-preinfected mice requires the production of type I IFNs but is independent of IFN-γ (Kudva et al., 2011; Lee et al., 2015). IFN-γ is primarily produced during the adaptive immune response by NK cells, activated CD8 cells, and Th1 cells, whereas type I IFNs are normally produced by innate immune cells at the early stage of infection. Type I IFNs may mediate Th17 cell differentiation during the primary T-cell response to bacteria as naive specific pathogen-free mice have not established memory response to primary bacterial infection.

The mechanisms underlying the IFN-γ-mediated attenuation of memory Th17 responses are mostly unknown. The IFN-γ-mediated downregulation of the expression of the chemokine responsible for Th17 cell trafficking to infected lungs (CCL17) may reflect an IFN-γ regulation of T cells in the context of the more complex environment of inflammation. The underlying mechanisms will be further identified in our future studies.

Secondary bacterial infection in the lungs is also associated with viruses other than IAV (Li et al., 2015; McCullers, 2014) and occurs in different mucosal sites, such as the middle ear and genitourinary tract (Brockson et al., 2012; Marom et al., 2012; Smith and McCullers, 2014; Torcia, 2019). IFN-γ expression is commonly induced during viral infections (Le Nouen et al., 2010), and Th17 is the primary T-cell subtype activated for bacterial clearance at mucosal sites. Hence, a defect in the IMR might be a common mechanism that contributes markedly to impaired bacterial clearance in the pathogenesis of viral-bacterial coinfection. Targeting this mechanism may be a valuable addition to the treatment options for refractory bacterial infections at mucosal sites.

The IFN-γ-mediated regulation of memory Th17 cells may not be involved in the reduced efficacy of pneumococcal vaccines because Th17 cells are not primarily induced through the intramuscular route used for the administration of such vaccines. However, our results revealed that the memory antibody response to coinfected *Sp* is impaired, which is consistent with the partially reduced humoral immunity against the bacteria following *Sp* vaccination in coinfected mice. Future studies are needed to clarify the underlying mechanisms so as to overcome the IAV-induced defects in the efficacy of the bacterial vaccine.

### Limitations of the Study

Although we demonstrated that IFN-γ impaired memory Th17 response to bacterial infection through inhibition of Th17 proliferation and migration and ruled out the role of IDO in suppressing Th17 responses in a model of an autoimmune disease, how IFN-γ causes the inhibition was not studied.

### Resource Availability

#### Lead Contact

Further information and requests for resources and reagents should be directed to and will be fulfilled by the Lead Contact, Beinan Wang (wangbn@im.ac.cn).

#### Materials Availability

This study did not generate new unique reagents.

#### Data and Code Availability

No data sets or code were generated or analyzed in this study. The raw data supporting the current study are available from the Lead Contact upon request. All software is commercially available.

## Methods

All methods can be found in the accompanying Transparent Methods supplemental file. The antibodies, organisms/strains, viral and bacterial strains, recombinant proteins, chemicals, critical commercial assays, oligonucleotides, and main software used in this study are listed in Table S1.

## Supplemental Information

Document S1. Transparent Methods, Figures S1–S6, and Table S1

## References

[bib1] Beshara R., Sencio V., Soulard D., Barthelemy A., Fontaine J., Pinteau T., Deruyter L., Ismail M.B., Paget C., Sirard J.C. (2018). Alteration of Flt3-Ligand-dependent de novo generation of conventional dendritic cells during influenza infection contributes to respiratory bacterial superinfection. PLoS Pathog..

[bib2] Brockson M.E., Novotny L.A., Jurcisek J.A., McGillivary G., Bowers M.R., Bakaletz L.O. (2012). Respiratory syncytial virus promotes Moraxella catarrhalis-induced ascending experimental otitis media. PLoS One.

[bib3] Bromley S.K., Mempel T.R., Luster A.D. (2008). Orchestrating the orchestrators: chemokines in control of T cell traffic. Nat. Immunol..

[bib4] Brundage J.F. (2006). Interactions between influenza and bacterial respiratory pathogens: implications for pandemic preparedness. Lancet Infect. Dis..

[bib5] Caucheteux S.M., Hu-Li J., Mohammed R.N., Ager A., Paul W.E. (2017). Cytokine regulation of lung Th17 response to airway immunization using LPS adjuvant. Mucosal Immunol..

[bib6] Duvigneau S., Sharma-Chawla N., Boianelli A., Stegemann-Koniszewski S., Nguyen V.K., Bruder D., Hernandez-Vargas E.A. (2016). Hierarchical effects of pro-inflammatory cytokines on the post-influenza susceptibility to pneumococcal coinfection. Sci. Rep..

[bib7] Fu H., Wang A., Mauro C., Marelli-Berg F. (2013). T lymphocyte trafficking: molecules and mechanisms. Front. Biosci. (Landmark Ed.).

[bib8] Groom J.R. (2019). Regulators of T-cell fate: integration of cell migration, differentiation and function. Immunol. Rev..

[bib9] Harada T., Ishimatsu Y., Hara A., Morita T., Nakashima S., Kakugawa T., Sakamoto N., Kosai K., Izumikawa K., Yanagihara K. (2016). Premedication with clarithromycin is effective against secondary bacterial pneumonia during influenza virus infection in a pulmonary emphysema mouse model. J. Pharmacol. Exp. Ther..

[bib10] Harrington L.E., Hatton R.D., Mangan P.R., Turner H., Murphy T.L., Murphy K.M., Weaver C.T. (2005). Interleukin 17-producing CD4+ effector T cells develop via a lineage distinct from the T helper type 1 and 2 lineages. Nat. Immunol..

[bib11] Janeway C.A. (2001). How the immune system works to protect the host from infection: a personal view. Proc. Natl. Acad. Sci. U S A.

[bib12] Kaka A.S., Foster A.E., Weiss H.L., Rooney C.M., Leen A.M. (2008). Using dendritic cell maturation and IL-12 producing capacity as markers of function: a cautionary tale. J. Immunother..

[bib13] Krummel M.F., Bartumeus F., Gerard A. (2016). T cell migration, search strategies and mechanisms. Nat. Rev. Immunol..

[bib14] Kudva A., Scheller E.V., Robinson K.M., Crowe C.R., Choi S.M., Slight S.R., Khader S.A., Dubin P.J., Enelow R.I., Kolls J.K. (2011). Influenza A inhibits Th17-mediated host defense against bacterial pneumonia in mice. J. Immunol..

[bib15] Le Nouen C., Hillyer P., Munir S., Winter C.C., McCarty T., Bukreyev A., Collins P.L., Rabin R.L., Buchholz U.J. (2010). Effects of human respiratory syncytial virus, metapneumovirus, parainfluenza virus 3 and influenza virus on CD4+ T cell activation by dendritic cells. PLoS One.

[bib16] Lee B., Gopal R., Manni M.L., McHugh K.J., Mandalapu S., Robinson K.M., Alcorn J.F. (2017). STAT1 is required for suppression of type 17 immunity during influenza and bacterial superinfection. Immunohorizons.

[bib17] Lee B., Robinson K.M., McHugh K.J., Scheller E.V., Mandalapu S., Chen C., Di Y.P., Clay M.E., Enelow R.I., Dubin P.J. (2015). Influenza-induced type I interferon enhances susceptibility to gram-negative and gram-positive bacterial pneumonia in mice. Am. J. Physiol. Lung Cell Mol. Physiol..

[bib18] Lee J., Lee J., Park M.K., Lim M.A., Park E.M., Kim E.K., Yang E.J., Lee S.Y., Jhun J.Y., Park S.H. (2013). Interferon gamma suppresses collagen-induced arthritis by regulation of Th17 through the induction of indoleamine-2,3-deoxygenase. PLoS One.

[bib19] Li N., Ren A., Wang X., Fan X., Zhao Y., Gao G.F., Cleary P., Wang B. (2015). Influenza viral neuraminidase primes bacterial coinfection through TGF-beta-mediated expression of host cell receptors. Proc. Natl. Acad. Sci. U S A.

[bib20] Li W., Moltedo B., Moran T.M. (2012). Type I interferon induction during influenza virus infection increases susceptibility to secondary Streptococcus pneumoniae infection by negative regulation of gammadelta T cells. J. Virol..

[bib21] Li Y., Yu X., Ma Y., Hua S. (2019). IL-23 and dendritic cells: what are the roles of their mutual attachment in immune response and immunotherapy?. Cytokine.

[bib22] Marom T., Nokso-Koivisto J., Chonmaitree T. (2012). Viral-bacterial interactions in acute otitis media. Curr. Allergy Asthma Rep..

[bib23] Matsuo K., Itoh T., Koyama A., Imamura R., Kawai S., Nishiwaki K., Oiso N., Kawada A., Yoshie O., Nakayama T. (2016). CCR4 is critically involved in effective antitumor immunity in mice bearing intradermal B16 melanoma. Cancer Lett..

[bib24] McCullers J.A. (2014). The co-pathogenesis of influenza viruses with bacteria in the lung. Nat. Rev. Microbiol..

[bib25] McCullers J.A., Rehg J.E. (2002). Lethal synergism between influenza virus and Streptococcus pneumoniae: characterization of a mouse model and the role of platelet-activating factor receptor. J. Infect. Dis..

[bib26] McNamee L.A., Harmsen A.G. (2006). Both influenza-induced neutrophil dysfunction and neutrophil-independent mechanisms contribute to increased susceptibility to a secondary Streptococcus pneumoniae infection. Infect. Immun..

[bib27] Metzger D.W., Furuya Y., Salmon S.L., Roberts S., Sun K. (2015). Limited efficacy of antibacterial vaccination against secondary serotype 3 pneumococcal pneumonia following influenza infection. J. Infect. Dis..

[bib28] Mikhak Z., Strassner J.P., Luster A.D. (2013). Lung dendritic cells imprint T cell lung homing and promote lung immunity through the chemokine receptor CCR4. J. Exp. Med..

[bib29] Morens D.M., Taubenberger J.K., Fauci A.S. (2008). Predominant role of bacterial pneumonia as a cause of death in pandemic influenza: implications for pandemic influenza preparedness. J. Infect. Dis..

[bib30] Nakae S., Iwakura Y., Suto H., Galli S.J. (2007). Phenotypic differences between Th1 and Th17 cells and negative regulation of Th1 cell differentiation by IL-17. J. Leukoc. Biol..

[bib31] Nakamura S., Davis K.M., Weiser J.N. (2011). Synergistic stimulation of type I interferons during influenza virus coinfection promotes Streptococcus pneumoniae colonization in mice. J. Clin. Invest..

[bib32] Park H.S., Costalonga M., Reinhardt R.L., Dombek P.E., Jenkins M.K., Cleary P.P. (2004). Primary induction of CD4 T cell responses in nasal associated lymphoid tissue during group A streptococcal infection. Eur. J. Immunol..

[bib33] Ramos-Sevillano E., Ercoli G., Brown J.S. (2019). Mechanisms of naturally acquired immunity to Streptococcus pneumoniae. Front. Immunol..

[bib34] Rathore J.S., Wang Y. (2016). Protective role of Th17 cells in pulmonary infection. Vaccine.

[bib35] Robinson K.M., Kolls J.K., Alcorn J.F. (2015). The immunology of influenza virus-associated bacterial pneumonia. Curr. Opin. Immunol..

[bib36] Robinson K.M., McHugh K.J., Mandalapu S., Clay M.E., Lee B., Scheller E.V., Enelow R.I., Chan Y.R., Kolls J.K., Alcorn J.F. (2014). Influenza A virus exacerbates Staphylococcus aureus pneumonia in mice by attenuating antimicrobial peptide production. J. Infect. Dis..

[bib37] Shahangian A., Chow E.K., Tian X., Kang J.R., Ghaffari A., Liu S.Y., Belperio J.A., Cheng G., Deng J.C. (2009). Type I IFNs mediate development of postinfluenza bacterial pneumonia in mice. J. Clin. Invest..

[bib38] Smith A.M., Huber V.C. (2018). The unexpected impact of vaccines on secondary bacterial infections following influenza. Viral Immunol..

[bib39] Smith A.M., McCullers J.A. (2014). Secondary bacterial infections in influenza virus infection pathogenesis. Curr. Top. Microbiol. Immunol..

[bib40] Stolberg V.R., Chiu B.C., Schmidt B.M., Kunkel S.L., Sandor M., Chensue S.W. (2011). CC chemokine receptor 4 contributes to innate NK and chronic stage T helper cell recall responses during Mycobacterium bovis infection. Am. J. Pathol..

[bib41] Sun K., Metzger D.W. (2008). Inhibition of pulmonary antibacterial defense by interferon-gamma during recovery from influenza infection. Nat. Med..

[bib42] Torcia M.G. (2019). Interplay among vaginal microbiome, immune response and sexually transmitted viral infections. Int. J. Mol. Sci..

[bib43] Tufail S., Badrealam K.F., Sherwani A., Gupta U.D., Owais M. (2013). Tissue specific heterogeneity in effector immune cell response. Front. Immunol..

[bib44] van Beelen A.J., Zelinkova Z., Taanman-Kueter E.W., Muller F.J., Hommes D.W., Zaat S.A., Kapsenberg M.L., de Jong E.C. (2007). Stimulation of the intracellular bacterial sensor NOD2 programs dendritic cells to promote interleukin-17 production in human memory T cells. Immunity.

[bib45] Wang B., Dileepan T., Briscoe S., Hyland K.A., Kang J., Khoruts A., Cleary P.P. (2010). Induction of TGF-beta1 and TGF-beta1-dependent predominant Th17 differentiation by group A streptococcal infection. Proc. Natl. Acad. Sci. U S A.

[bib46] Zha Z., Bucher F., Nejatfard A., Zheng T., Zhang H., Yea K., Lerner R.A. (2017). Interferon-gamma is a master checkpoint regulator of cytokine-induced differentiation. Proc. Natl. Acad. Sci. U S A.

