# Supplementary material for: Flu Virus Attenuates Memory Clearance of Pneumococcus via IFN-γ-Dependent Th17 and Independent Antibody Mechanisms
Source: iScience. 2020 Nov 4;23(12):101767. doi: 10.1016/j.isci.2020.101767 (PMC7683269; doi:10.1016/j.isci.2020.101767)
Supplement: Document S1. Transparent Methods, Figures S1–S6, and Table S1 [file mmc1.pdf]

iScience, Volume *23*

## Supplemental Information

# Flu Virus Attenuates Memory Clearance of *Pneumococcus* via IFN-$\gamma$-Dependent Th17 and Independent Antibody Mechanisms

**Ning Li, Xin Fan, Meiyi Xu, Ya Zhou, and Beinan Wang**

**Supplemental Information**

# Influenza A Virus Attenuates Memory Clearance of *Streptococcus pneumoniae via* Restriction of IFN-γ Dependent Th17 and Independent Antibody Responses

Ning Li, Xin Fan, Meiyi Xu, Ya Zhou, and Beinan Wang

## Transparent Methods

### Ethics statement

This study was carried out in strict accordance with the recommendations in the *Guide for the Care and Use of Laboratory Animals* of the Institute of Microbiology, Chinese Academy of Sciences (IMCAS, Beijing, China). The study protocol was approved by the Committee on the Ethics of Animal Experiments of IMCAS (APIMCAS2016025). Mice were bred under SPF conditions in the Laboratory Animal Facility at IMCAS. Animal experiments were conducted under isoflurane anesthesia, and all efforts were made to minimize their suffering.

### Viral and bacterial strains

A/Puerto Rico/08/1934 (PR8) virus (the mouse-adapted H1N1 influenza-A virus) was cultured in the allantoic cavities of 9-day-old SPF embryonated hen eggs and incubated for 2 days at 35 °C. The allantoic fluid was collected and stored at −80 °C. Viruses were quantified in Madin–Darby canine kidney cells and expressed as 50% tissue culture infective dose ($TCID_{50}$). *Sp strain* ST556 (serotype-19F) is a clinical isolate that can cause bacteremia (Li et al., 2015). $GAS^{OVA}$ (a group-A streptococcus strain (90-226) genetically engineered to express the ovalbumin peptide (amino acids 323–339) on bacteria surfaces) was a kind gift from Dr. P. P. Cleary (University of Minnesota, Minneapolis, MN, USA) (Park et al., 2004). These bacteria were maintained on sheep-blood agar and grown in THY-Neo (Todd–Hewitt broth supplemented with 2% neopeptone; BD Bioscience, San Jose, CA, USA) medium at

37 ℃ in an atmosphere of 5% $CO_2$. Overnight cultures were harvested at the optical density (O.D 560 nm) reaching to ~ 1.1 and used for mouse infection. Colony-forming units (CFUs) were verified by plating on blood agar.

**Mice and infection**

Female C57BL/6J and CD45.1 mice (B6.SJL-*Ptprc*$^{a}$ *Pepc*$^{b}$/BoyJ) (6–8 weeks) were purchased from Vital River Laboratories Animal Center (Beijing, China). *Ifng*$^{-/-}$ mice (B6.129S7-*Ifng*$^{tm1Ts}$/J) were obtained from the Jackson Laboratory (Bar Harbor, ME, USA). OT-Ⅱ transgenic mice (B6.Cg-Tg (TcraTcrb) 425Cbn/J) were a kind gift from Dr. B. Hou (Institute of Biophysics, Chinese Academy of Sciences, Beijing, China) and backcrossed for ≥6 generations to B6 mice in the animal facility of the Institute of Microbiology of Chinese Academy of Sciences followed by intercrossing to produce homozygous offspring. Genotypes of offspring mice were determined by PCRs of tail DNA provided by the Jackson Laboratory. Those mice were of a C57BL/6 background, and control C57BL/6 mice were age- and sex-matched when used. Mice were anesthetized (sodium pentobarbital) and inoculated intratracheally (i.t.) with *Sp* strain ST556 ($3.0\times10^{7}$/mouse) in 30 μL of PBS. Four weeks later, the mice were anesthetized, infected (i.t.) with PR8 at 30 $TCID_{50}$ in 30 μL of PBS, and challenged with the same dose of *Sp* 7 days after PR8 infection. Five days after challenge, lung tissue was collected for CFU counting and flow cytometry (Fan et al., 2014). Single-cell suspensions of HLNs and/or lungs were prepared for flow cytometry analyses. Blood taken by cardiac puncture and

lung-homogenate supernatants were collected and stored at −20 °C until ELISAs were undertaken. For survival assays, *Sp*-preinfected mice were infected with PR8 as before and challenged with a lethal dose of *Sp* ST556 ($1\times10^8$/mouse) 7 days after PR8 infection. Weight loss and survival of infected mice were documented once a day over a 15-day period. In addition to mice that were found dead, mice with 30% loss of their starting bodyweight were euthanized and recorded as dead. The outcome of mortality was anticipated and approved by the Animal Ethics Committee of our institution. At the end of the experiment, surviving mice were euthanized by exposure to increasing concentrations of $CO_2$.

**Splenocyte co-culture with HK-*Sp***

Four weeks after *Sp* infection, spleens from *Ifng*$^{-/-}$ mice or WT mice were removed and single-cell suspensions prepared in RPM1 1640 (Gibco, Grand Island, NY, USA) supplemented with antibiotics and 10% fetal bovine serum (Gibco). After removing red blood cells with ACK lysis buffer (2.06% Tris, pH 7.65, and 0.83% $NH_4Cl$) the cell suspension was passed through a 40-mm strainer (BD Falcon, Bedford, MA, USA) and plated on a 48-well, flat-bottomed plate ($1\times10^6$, 500 µL/well). Cells were stimulated with HK-*Sp* (multiplicity of infection = 10) with recombinant mouse IFN-γ (rmIFN-γ; GenScript, Piscataway, NJ, USA) at different concentrations for 7 days. The culture supernatant was collected for IL-17 measurement by ELISAs.

## IFN-γ treatment

Mice were preinfected with *Sp* ($3.0\times10^7$/mouse) and injected (i.v.) with 50 ng of rmIFN-γ (GenScript) in 100 μL of PBS and injected (i.t.) with 2 μg of rmIFN-γ (GenScript) in 25 μL of PBS containing 1% mouse serum or only 1% mouse serum in PBS as a control every other day for five doses. Thirty-five days after *Sp* preinfection, mice were challenged with *Sp* ($3\times10^7$/mouse) and euthanized 5 days following this challenge to determine the number of Th17 cells in lungs and IL-17 production in lung-homogenate supernatants.

## Neutralization of IL-17 *in vivo*

Mice were preinfected with *Sp* ($3\times10^7$/mouse) followed by infection with the PR8 (30 $TCID_{50}$/mouse) as described in the "mice and infection" section. After PR8 infection, the mice were injected intraperitoneal either with 100 μg of an IL-17 antibody (17F3; BioXCell, Lebanon, New Hampshire, USA) to block IL-17 or with an isotype control (MOPC-21; BioXCell) every other day. The mice were challenged with *Sp* ($3\times10^7$/mouse) 7 days after the PR8 infection and euthanized 5 days following the challenge to determine the number of CFUs in the lungs.

## 1-Methyl-D-tryptophan (D-1MT) treatment

D-1MT (452483; Sigma–Aldrich, Saint Louis, MO, USA) was prepared as a 20-mg/ml stock solution in 0.1 M NaOH and protected from light. Mice were preinfected with *Sp* ($3\times10^7$/mouse) followed by infection with PR8 (30 $TCID_{50}$/mouse)

as described in the "mice and infection" section. Three days before PR8 infection, the mice were administered either D-1MT (2 mg/ml) in drinking water containing sweetener (Nutrasweet) to enhance palatability or drinking solvent as a control (Baban et al., 2009). The mice were challenged with *Sp* ($3 \times 10^7$/mouse) 7 days after the PR8 infection and euthanized 5 days later to determine the numbers of CFUs and Th17 cells in the lungs.

**Adoptive cell transfer**

Recipient mice (CD45.1) were inoculated with PR8 or PBS 6 days before cell transfer. Donor $CD4^+$ T cells were isolated from the spleen and lymph nodes of $GAS^{OVA}$-infected mice or naive OT-Ⅱ transgenic mice (CD45.2) with anti-CD4 microbeads (Miltenyi Biotec, Bergisch Gladbach, Germany) according to manufacturer instructions. Donor cells were labeled with 1 μM of CFSE (Sigma–Aldrich) for 5 min at 37 ℃, and $2 \times 10^6$ of these cells were transferred to each recipient by tail-vein injection. Twenty-four hours after transfer, recipient mice were challenged with $GAS^{OVA}$ ($2 \times 10^7$/mouse) and euthanized 3 days after challenge for flow cytometry analyses in HLNs and lungs (Caucheteux et al., 2017).

**ELISA**

IL-17 (IL-17A) production was determined using a Ready-SET-Go!® ELISA kit (eBioscience, San Diego, CA, USA) according to manufacturer instructions. Levels of *Sp*-specific antibody were measured by endpoint ELISA. Briefly, 96-well plates

(Corning Costar, Corning, NY, USA) were coated with HK-*Sp*. Samples (100 μL) were added and incubated for 2 h at 37 ℃. Horseradish peroxidase-conjugated goat anti-mouse IgG antibody (Southern Biotech, Birmingham, AL, USA) was used as the secondary antibody (1:4000 dilution). The reaction was developed by addition of 3,3',5,5'-Tetramethylbenzidine (TMB) (Tiangen Biotech, Beijing, China) and measured by an ELx800 plate reader (BioTek, VT, USA) at 450 nm with an absorbance at 570 nm as an internal control. A standard curve was generated by adding twofold-diluted mouse IgG (Biovision, Martin View, CA, USA). The IgG concentration was calculated based on the standard curve.

**Cellular staining and flow cytometry**

Single-cell suspensions of HLNs and lungs were prepared in flow cytometry buffer (PBS with 0.01% $NaN_3$ and 0.2% bovine serum albumin). Cell staining and flow cytometry for T cells were conducted as follows. For staining of intracellular cytokines, cells were stimulated with phorbol 12-myristate 13-acetate (PMA) and ionomycin, and treated with brefeldin A. Cells were stained for surface markers with anti-CD3 (145-2C11; BioLegend, San Diego, CA), anti-CD4 (GK1.5; eBiosciences), anti-CD11c (N418; eBiosciences), anti-MHC Ⅱ (M5/114.15.2; eBiosciences), anti-CD80 (16-10A1; BioLegend), anti-CD86 (GL1; eBiosciences), anti-CD8α (53-6.7; eBiosciences), anti-NK-1.1 (PK136; BioLegend), anti-CD45.2 (104; BioLegend), and anti-CCR4 (2G12; BioLegend). For intracellular staining, fixed cells were permeabilized and stained with IL-17A (eBio17B7; eBiosciences) and

anti-RORγt (B2D; eBioscience) for Th17 cells and IFN-γ (XMG1.2; BioLegend) for Th1 cells (Wang et al., 2017). Samples were tested on a FACSAria Ⅱ flow cytometer (BD Biosciences) and analyzed by FlowJo (Tree Star, Ashland, OR, USA).

**Immunohistochemistry**

Lung lobes were fixed in 4% paraformaldehyde and embedded in paraffin. Immunohistochemistry was carried out as follows. Sections were blocked with 10% fetal bovine serum in PBS followed by staining with anti- CCL17 antibody (Sigma–Aldrich), incubated with biotinylated anti-rabbit antibody (Santa Cruz Biotechnology, Santa Cruz , CA, USA) followed by an avidin-enzyme complex. CCL17 expression was visualized with 3,3′-Diaminobenzidine (Sigma–Aldrich) and nuclei were counterstained with hematoxylin. PBS was used for washing after each step. Slides were mounted in permanent mounting media (Dako, Glostrup, Denmark) and digitally scanned using Pannoramic SCAN slice-scanner (3D-Histech, Budapest, Hungary) (Mikhak et al., 2013). The positive rate of CCL17 expression cells was assessed quantitatively with Imagepro Plus image analysis software (Zhang et al., 2013).

**RNA extraction and quantitative real-time polymerase chain reaction (qPCR)**

Total RNA was extracted from lung tissue with TRIzol® Reagent (Invitrogen, Carlsbad, CA, USA). Reverse transcription was carried out using High Capacity cDNA Reverse Transcription Kit (Thermo Fisher Scientific, Waltham, MA, USA) according to

manufacturer instructions. Transcripts were amplified with SYBR Premix Ex Taq™ Ⅱ (TaKaRa Biotechnology, Shiga, Japan) on a 480 Ⅱ system (Roche, Basel, Switzerland) using specific primer sets. Relative expression was evaluated using the $2^{-\Delta\Delta Ct}$ method and expression of glyceraldehyde 3-phosphate dehydrogenase (GAPDH) was used as the internal control. qPCR primers (forward and reverse, respectively) were ordered from Invitrogen and used for mouse genes: CCL17, 5′-CAGGGATGCCATCGTGTTTC-3′ and 5′- CACCAATCTGATGGCCTTCTT-3′; GAPDH, 5′-CATGGCCTTCCGTGTTCCTA-3′ and 5′-GCGGCACGTCAGATCCA-3′.

## Statistical analyses

Statistical analyses were undertaken using one-way ANOVA, followed by Tukey's multiple comparisons test for comparison of three or more groups of sample data. The log-rank test was employed to measure survival. The 2-tailed unpaired Mann-Whitney *U* nonparametric *t*-test was used for CFUs. 2-tailed unpaired *t*-test was employed for other variables using Prism v8.0 (GraphPad, San Diego, CA, USA). $P \leq 0.05$ was considered significant.

**Table S1. Key Resources Table.** Related to Figures 1-7.

| REAGENT OR RESOURCE | SOURCE | IDENTIFIER |
|---|---|---|
| **Antibodies** | | |
| Goat anti-Mouse IgG-Fc Fragment Antibody | Bethyl | Cat#A90-131A |
| Goat Anti-Mouse IgG, Human ads-HRP | Southern Biotech | Cat#1030-05 |
| Mouse IgG | BioVision | Cat#1265-100 |
| Anti-Mouse CD3 (17A2) FITC | eBiosciences | Cat#11-0032-82 |

| | | |
|---|---|---|
| Anti-Mouse CD3 (145-2C11) PerCP | BioLegend | Cat#100325 |
| Anti-Mouse CD4 (GK1.5) FITC | eBiosciences | Cat#11-0041-85 |
| Anti-Mouse CD4 (GK1.5) PerCP | BioLegend | Cat#100432 |
| Anti-Mouse CD4 (GK1.5) PE-Cyanine7 | eBiosciences | Cat#25-0041-81 |
| Anti-Mouse CD8α (53-6.7) FITC | eBiosciences | Cat#11-0081-82 |
| Anti-Mouse NK-1.1 (PK136) PE | BioLegend | Cat#108707 |
| Anti-Mouse CD11c (N418) PerCP-Cyanine5.5 | eBiosciences | Cat#45-0114-80 |
| Anti-Mouse MHC Ⅱ(M5/114.15.2) FITC | eBiosciences | Cat#11-5321-81 |
| Anti-Mouse CD80 (16-10A1) PE | BioLegend | Cat#104707 |
| Anti-Mouse CD86 (GL1) APC | eBiosciences | Cat#17-0862-81 |
| Anti-Mouse CD45.2 (104) PE | BioLegend | Cat#109808 |
| Anti-Mouse CCR4 (2G12) | BioLegend | Cat#131211 |
| Anti-Mouse IL-17A (eBio17B7) FITC | eBioscience | Cat#11-7177-81 |
| Anti-Mouse IL-17A (eBio17B7) APC | eBioscience | Cat#17-7177-81 |
| Anti-Mouse RORγt (B2D) APC | eBioscience | Cat#14-6981-80 |
| Anti-Mouse IFN-γ (XMG1.2) PE | BioLegend | Cat#505807 |
| Anti-Mouse IFN-γ (XMG1.2) APC | eBioscience | Cat#17-7311-81 |
| **Experimental Models: Organisms/Strains** | | |
| Mouse: C57BL/6J | Jackson Laboratory | Stock#000664 |
| Mouse: B6.SJL-Ptprc$^{a}$ Pepc$^{b}$/BoyJ | Jackson Laboratory | Stock#002014 |
| Mouse: B6.129S7-Ifng$^{tm1Ts}$/J | Jackson Laboratory | Stock#002287 |
| Mouse: B6.Cg-Tg (TcraTcrb) 425Cbn/J | Jackson Laboratory | Stock# 004194 |
| **Experimental Models: Viral and Bacterial strains** | | |
| A/Puerto Rico/08/1934 (PR8) virus | saved in our laboratory | (Li et al., 2015) |
| *Sp* strain ST556 | clinical isolate | (Li et al., 2015) |
| GAS$^{OVA}$ | Dr. P. P. Cleary University of Minnesota, Minneapolis, MN, USA | (Park et al., 2004) |
| **Recombinant proteins** | | |
| recombinant mouse IFN-γ | GenScript | Cat#Z02916-500 |
| **Chemicals** | | |
| Todd–Hewitt broth | BD | Cat#249240 |
| Neopeptone | BD | Cat#211681 |
| CFSE | Sigma–Aldrich | Cat#21888-25mg-F |
| Soluble TMB Substrate Solution | Tiangen Biotech | Cat#PA107-01 |
| Ionomycin | Sigma | Cat#I0634-1MG |
| phorbol 12-myristate 13-acetate (PMA) | Sigma | Cat#P1585-1MG |

| | | |
|---|---|---|
| Brefeldin A | eBioscience | Cat#00-4506-51 |
| 1-methyl-D-tryptophan (D-1MT) | Sigma–Aldrich | Cat# 452483-1G |
| **Critical Commercial Assays** | | |
| CD4(L3T4) Microbeads, mouse | Miltenyi Biotec | Cat#130-117-043 |
| Foxp3 / Transcription Factor Staining Buffer Set Kit | eBioscience | Cat#00-5523-00 |
| Mouse IL-17A (homodimer) ELISA kit | eBioscience | Cat#88-7371-88 |
| TRIZOL | Invitrogen | Cat#15596-026 |
| High Capacity cDNA Reverse Transcription Kit | ThermoFisher Scientific | Cat#4368814 |
| SYBR Premix Ex Taq$^{TM}$ II | TaKaRa | Cat#RR820A |
| **Oligonucleotides** | | |
| CCL17 Forward CAGGGATGCCATCGTGTTTC | Invitrogen DNA Technologies | N/A |
| CCL17 Reverse CACCAATCTGATGGCCTTCTT | Invitrogen DNA Technologies | N/A |
| GAPDH Forward CATGGCCTTCCGTGTTCCTA | Invitrogen DNA Technologies | N/A |
| GAPDH Reverse GCGGCACGTCAGATCCA | Invitrogen DNA Technologies | N/A |
| **Software** | | |
| PRISM Version 8 | Graphpad | https://www.graphpad.com/scientific-software/prism/ |
| FlowJo Version 7 | Tree Star | https://www.flowjo.com/solutions/flowjo/downloads |
| Adobe Creative Cloud | Adobe | https://www.adobe.com/creativecloud.html# |

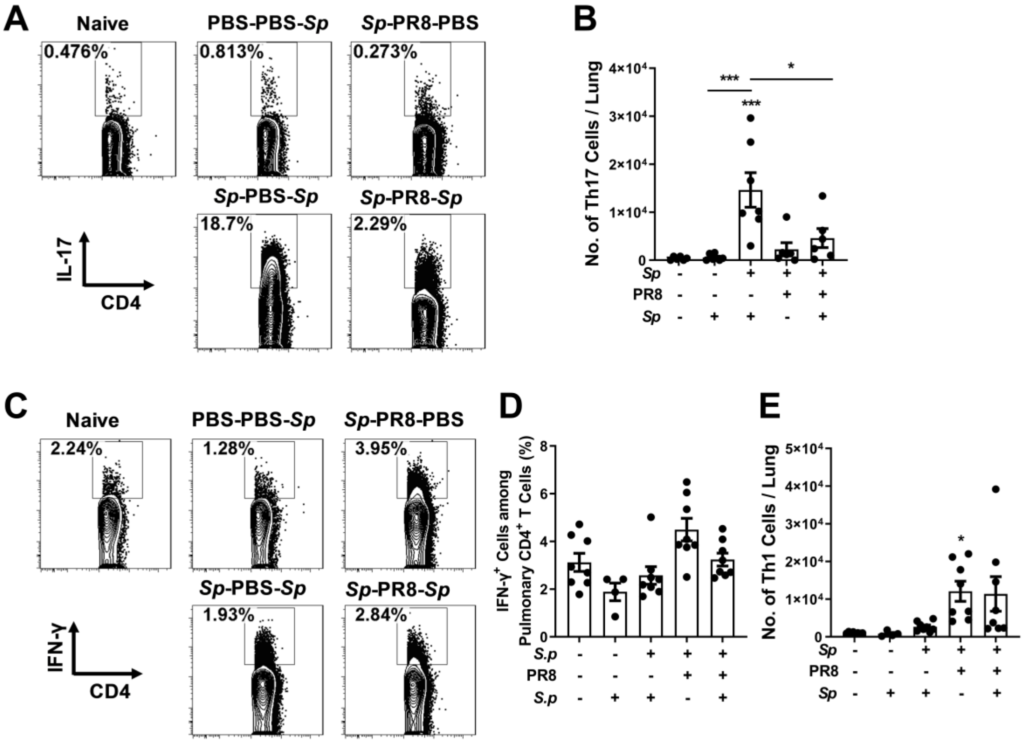


**Figure S1. Memory-mediated bacterial clearance was impaired in coinfected mice which showed a reduced Th17 cell response to secondary *Sp* infection.**

Related to Figure 1. Mice were infected, challenged, and euthanized as described in Figure 1B. The Lungs were removed for flow cytometry analyses. (A) Dot plots represent the expression of CD4 and IL-17 on gated $CD4^+$ T cells. (B) The number of $IL\text{-}17^+$ $CD4^+$ T cells in the lungs was determined by flow cytometry ($n$ = 4–8). (C) Dot plots represent the expression of CD4 and IFN-γ on gated $CD4^+$ T cells. (D) The proportion of $IFN\text{-}\gamma^+$ cells among $CD4^+$ T cell population and (E) the number of $IFN\text{-}\gamma^+$ $CD4^+$ T cells in the lungs were determined by flow cytometry ($n$ = 4–8). Data are represented as mean +/- SEM of 2–3 independent experiments. *$p < 0.05$, ***$p < 0.001$. (B, D, and E) One-way ANOVA, followed by Tukey's multiple comparisons test.

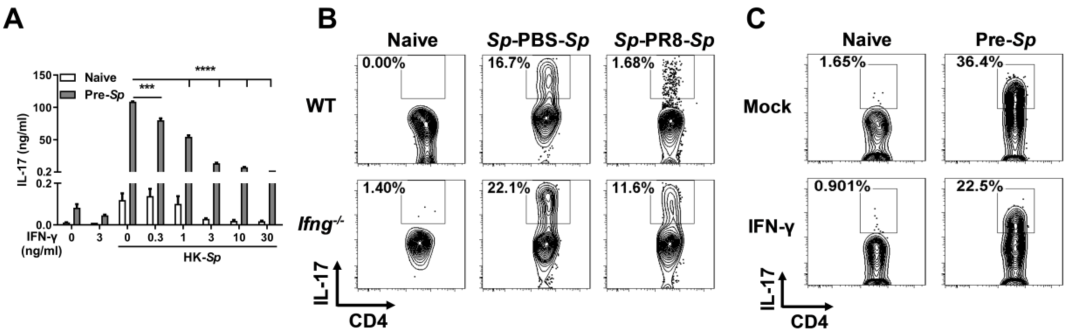


**Figure S2. IFN-γ deficiency rescued the response of memory Th17 cells to the bacteria in coinfected mice.** Related to Figure 3. (A) Mouse splenocytes were cultured with or without heat-killed *Streptococcus pneumoniae* (HK-*Sp*) and costimulated with the indicated concentration of recombinant mouse IFN-γ for 7 days ($n$ = 6). IL-17 concentration in the culture supernatants was measured by ELISA. Data are represented as mean +/- SEM of 2 independent experiments. ***$p < 0.001$, ****$p < 0.0001$. One-way ANOVA, followed by Tukey's multiple comparisons test. Mice were infected, challenged, and euthanized as described in (B) Figure 1B or (C) Figure 3F. The Lungs were removed for flow cytometry analyses. Dot plots represent the expression of CD4 and IL-17 on gated $CD4^+$ T cells.

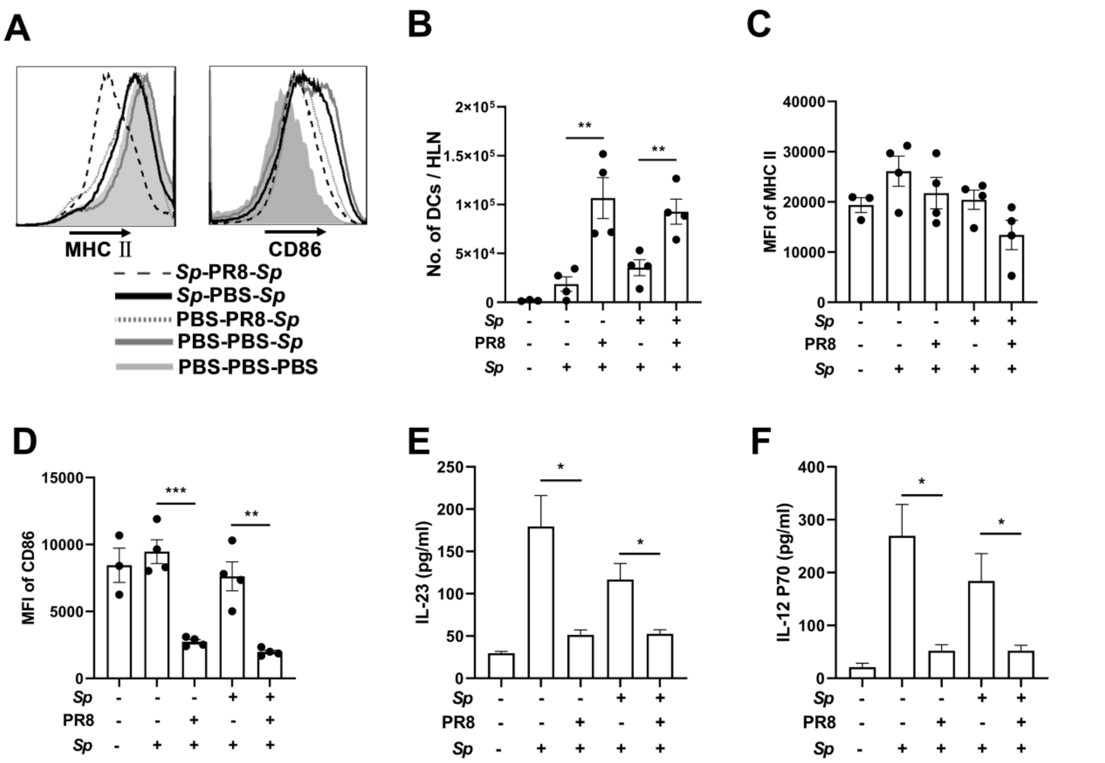


**Figure S3. The activation of DCs and IL-23 produced by DCs were both inhibited in IAV-infected mice independently of IFN-γ.** Related to Figure 4. Mice were infected and challenged as described in Figure 1B. Two days after *Streptococcus pneumoniae* (*Sp*) challenge, the mice were euthanized ($n = 4$). (A) Representative image of MHC class Ⅱ (left) and CD86 (right) expression on the surface of dendritic cells (DCs) in the lungs. (B) The number of $CD11c^+$ cells in hilar lymph nodes (HLNs) and the mean fluorescence intensity (MFI) for (C) MHC class Ⅱ and (D) CD86 on the surface of DCs in HLNs were determined by flow cytometry. (E) IL-23 and (F) IL-12p70 concentration in HLN homogenates was determined by ELISA. Data are represented as mean +/- SEM of 2 independent experiments. *$p < 0.05$, **$p < 0.01$, ***$p < 0.001$. (B-F) One-way ANOVA, followed by Tukey's multiple comparisons test.

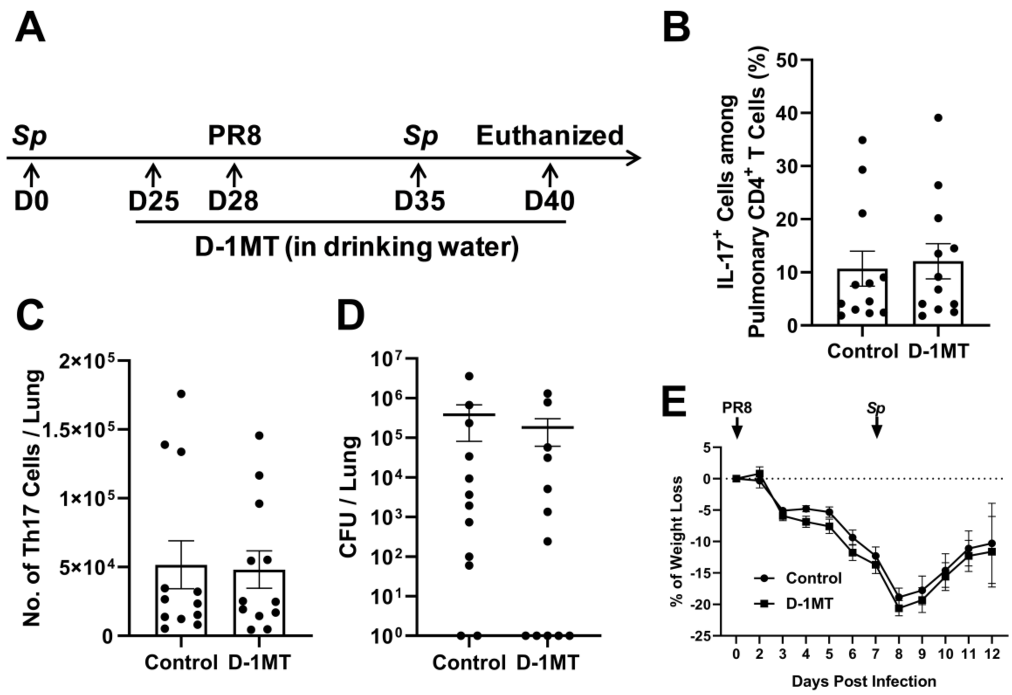


**Figure S4. The indoleamine 2,3-dioxygenase (IDO) pathway was not involved in the reduction in the Th17 cell responses during coinfection.** Related to Figure 4. (A) Schematic illustration of the experimental design for IDO inhibitor 1-Methyl-D-tryptophan (D-1MT) treatment and infection. Mice were infected and challenged as described in Figure 1B. Three days before infection with the influenza A virus PR8 strain, mice were administered either D-1MT (2 mg/ml) in drinking water or drinking solvent as a control. The mice were euthanized 5 days after *Streptococcus pneumoniae* (*Sp*) challenge. (B) The proportion of IL-$17^+$ cells among pulmonary $CD4^+$ T cell population and (C) the number of IL-$17^+$ $CD4^+$ T cells in the lungs were determined by flow cytometry ($n$ = 12). (D) The numbers of colony-forming units (CFUs) in the lungs were determined. ($n$ = 12). (E) Body weight was measured once daily from the day of PR8 infection until euthanasia ($n$ = 12). Data are represented as mean +/- SEM of 2–3 independent experiments. (B and C) Two-tailed unpaired *t*-test; (D) 2-tailed unpaired Mann-Whitney *U* nonparametric

*t*-test.

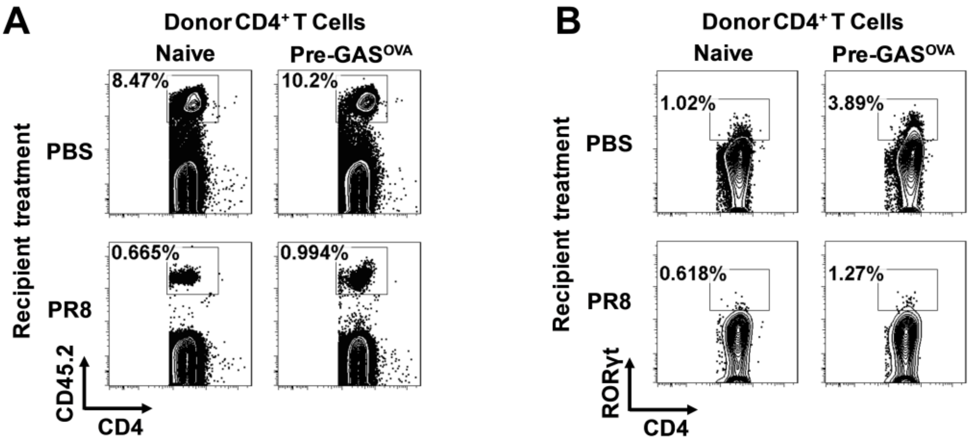


**Figure S5. The proliferation of Th17 cells in response to secondary Sp infection was suppressed in IAV-infected mice.** Related to Figure 5. The adoptive transfer was done as described in Figure 5A. Dot plots represent the expression of (A) CD4 and CD45.2 on gated lymphocytes and (B) CD4 and RORγt on gated $CD4^+$ $CD45.2^+$ T cells in the hilar lymph nodes (HLNs).

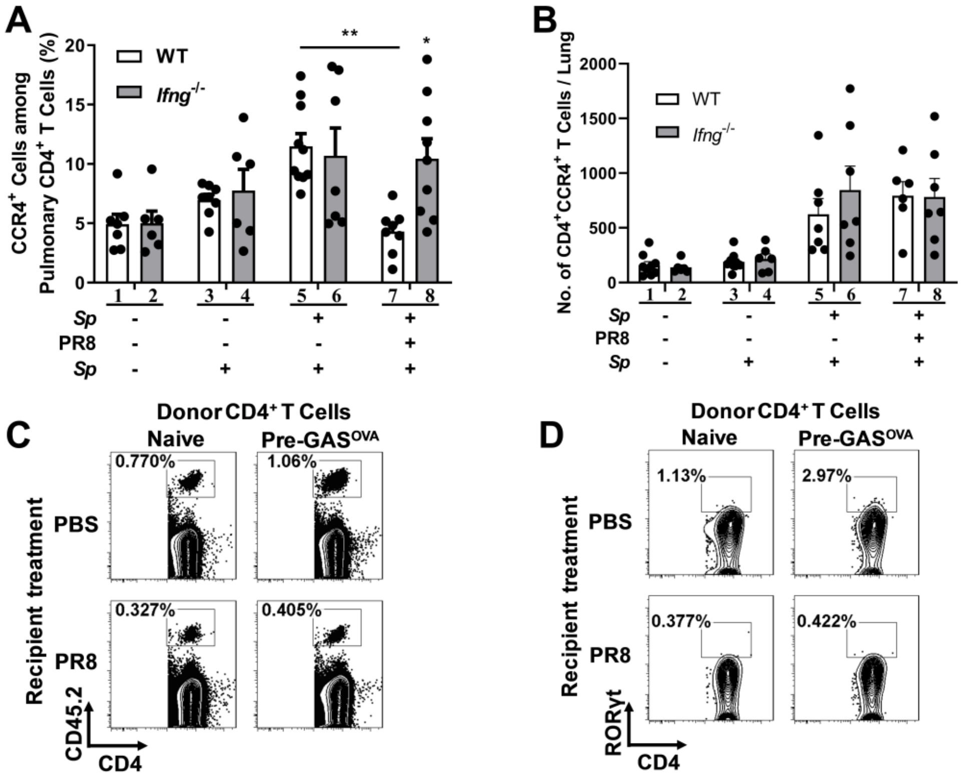


**Figure S6. The trafficking of Th17 cells to the lungs was delayed in coinfected mice in response to secondary *Sp* infection.** Related to Figure 6. Mice were infected, challenged, and euthanized as described in Figure 1B. (A) The proportion of $CCR4^+$ cells among the pulmonary $CD4^+$ T cell population and (B) the number of pulmonary $CCR4^+$ $CD4^+$ T cells were determined by flow cytometry ($n$ = 6–10). Data are represented as mean +/- SEM of 2–3 independent experiments. *$p < 0.05$, **$p < 0.01$. (A and B) One-way ANOVA, followed by Tukey's multiple comparisons test. The adoptive transfer was done as described in Figure 5A. Dot plots represent the expression of (C) CD4 and CD45.2 on gated lymphocytes and (D) CD4 and RORγt on gated $CD4^+$ $CD45.2^+$ T cells in the lungs.

## REFERENCES

Baban, B., Chandler, P.R., Sharma, M.D., Pihkala, J., Koni, P.A., Munn, D.H., and Mellor, A.L. (2009). IDO activates regulatory T cells and blocks their conversion into Th17-like T cells. Journal of immunology (Baltimore, Md : 1950) *183*, 2475-2483.

Caucheteux, S.M., Hu-Li, J., Mohammed, R.N., Ager, A., and Paul, W.E. (2017). Cytokine regulation of lung Th17 response to airway immunization using LPS adjuvant. Mucosal Immunol *10*, 361-372.

Fan, X., Wang, X., Li, N., Cui, H., Hou, B., Gao, B., Cleary, P.P., and Wang, B. (2014). Sortase A induces Th17-mediated and antibody-independent immunity to heterologous serotypes of group A streptococci. PloS one *9*, e107638.

Li, N., Ren, A., Wang, X., Fan, X., Zhao, Y., Gao, G.F., Cleary, P., and Wang, B. (2015). Influenza viral neuraminidase primes bacterial coinfection through TGF-beta-mediated expression of host cell receptors. Proc Natl Acad Sci U S A *112*, 238-243.

Mikhak, Z., Strassner, J.P., and Luster, A.D. (2013). Lung dendritic cells imprint T cell lung homing and promote lung immunity through the chemokine receptor CCR4. J Exp Med *210*, 1855-1869.

Park, H.S., Costalonga, M., Reinhardt, R.L., Dombek, P.E., Jenkins, M.K., and Cleary, P.P. (2004). Primary induction of CD4 T cell responses in nasal associated lymphoid tissue during group A streptococcal infection. Eur J Immunol *34*, 2843-2853.

Wang, X., Fan, X., Bi, S., Li, N., and Wang, B. (2017). Toll-like Receptors 2 and 4-Mediated Reciprocal Th17 and Antibody Responses to Group A Streptococcus Infection. The Journal of infectious diseases *215*, 644-652.

Zhang, X., Wang, H., and Wang, J. (2013). Expression of HMGB1 and NF-kappaB p65 and its significance in non-small cell lung cancer. Contemp Oncol (Pozn) *17*, 350-355.
